# A post-transcriptional respiratome regulon in trypanosomes

**DOI:** 10.1093/nar/gkz455

**Published:** 2019-05-25

**Authors:** Anna Trenaman, Lucy Glover, Sebastian Hutchinson, David Horn

**Affiliations:** The Wellcome Trust Centre for Anti-Infectives Research, School of Life Sciences, University of Dundee, Dow Street, Dundee DD1 5EH, UK

## Abstract

Post-transcriptional regulons coordinate the expression of groups of genes in eukaryotic cells, yet relatively few have been characterized. Parasitic trypanosomatids are particularly good models for studies on such mechanisms because they exhibit almost exclusive polycistronic, and unregulated, transcription. Here, we identify the *Trypanosoma brucei* ZC3H39/40 RNA-binding proteins as regulators of the respiratome; the mitochondrial electron transport chain (complexes I–IV) and the F_o_F_1_-ATP synthase (complex V). A high-throughput RNAi screen initially implicated both ZC3H proteins in variant surface glycoprotein (*VSG*) gene silencing. This link was confirmed and both proteins were shown to form a cytoplasmic ZC3H39/40 complex. Transcriptome and mRNA-interactome analyses indicated that the impact on *VSG* silencing was indirect, while the ZC3H39/40 complex specifically bound and stabilized transcripts encoding respiratome-complexes. Quantitative proteomic analyses revealed specific positive control of >20 components from complexes I, II and V. Our findings establish a link between the mitochondrial respiratome and *VSG* gene silencing in bloodstream form *T. brucei*. They also reveal a major respiratome regulon controlled by the conserved trypanosomatid ZC3H39/40 RNA-binding proteins.

## INTRODUCTION

Coordinated regulation of functionally related groups of genes greatly facilitates the ability of cells to rapidly respond to changes in environmental conditions. Prokaryotic cells typically display clustering of such genes into transcriptionally and translationally co-regulated ‘operons’. Seventeen years ago, Keene and Tenenbaum proposed an alternative post-transcriptional ‘operon’ model for nucleated cells, in which transcription and translation are compartmentalized (1). Several such ‘regulons’, under the control of regulatory RNA-binding proteins (RBPs), have subsequently been identified; with RBPs that operate through binding mRNA 3′ untranslated regions (UTRs) featuring particularly prominently (2). Although it is now widely accepted that post-transcriptional regulons make major contributions to coordinated gene expression control in eukaryotic cells, relatively few have been characterized in any detail. Indeed, a recent review focussing on the trypanosomatids highlights our ‘extremely limited understanding of the contributions of ... [RBPs] to mRNA fate’ (3).

The trypanosomatids are a group of related parasitic protozoa, several of which cause important and lethal diseases in humans and animals. They include *Trypanosoma brucei*, the causative agent of human African trypanosomiasis and nagana in cattle; *Trypanosoma cruzi*, which causes Chagas disease; and multiple *Leishmania* species, which cause diseases known as the leishmaniases (4). A unique feature of trypanosomatid biology, which makes them particularly good models for studies on post-transcriptional mechanisms of gene-expression control, is pervasive polycistronic transcription of almost all genes, with little evidence for clustering of functionally related groups of genes (5). Thus, regulated mRNA stability and translation, coordinated by RBPs, is particularly important in trypanosomatids; since regulated expression must operate almost exclusively post-transcription.

Codon usage has been shown to be a major contributor to differential gene expression in trypanosomatids and in other organisms, with more efficient translation of transcripts containing a majority of codons with highly abundant cognate tRNAs. Indeed, in trypanosomes, codon usage exhibits a strong correlation with mRNA and protein abundance and, in this case, translation also increases mRNA half-life (6,7). Although codon usage impacts global mRNA and protein abundance, regulatory RBPs add an important layer of additional control, facilitating differential expression and adaptation to the vastly different environments encountered during the trypanosome life cycle; through the midgut and salivary gland of the tsetse-fly vector to the blood and tissues of mammalian hosts in the case of *T. brucei*. Indeed, among hundreds of RBPs encoded in each trypanosomatid genome, those regulatory RBPs that have been characterized typically control gene expression changes that drive, or correlate with, developmental progression (reviewed in 3,8,9).

Post-transcriptional regulons may operate at the level of mRNA maturation, transport or stability, or at the level of protein translation (reviewed in 3,8,9). The regulatory RBPs are typically thought to bind to *cis*-acting elements within mRNA 3′-UTRs, mediated by specific sequence motifs or secondary structures. Indeed, this view is supported for trypanosomatids by *in silico* and *in vitro* analyses (10). One particular challenge, however, has been to identify specific cohorts of mRNAs bound by specific regulatory RBPs *in vivo*. The majority of studies also focus on mRNA abundance rather than protein abundance, meaning that translational control remains under-studied.

Regulatory *T. brucei* 3′-UTRs have been identified for genes encoding the hexose transporters (11), cytochrome oxidase subunits (12), protein associated with differentiation 1 (13) and the translationally controlled tumour protein orthologues (14); as well as the major surface antigens, the procyclins (15) and variant surface glycoproteins (VSGs) (16). Although the number of regulatory RBPs characterized in *T. brucei* has increased rapidly in recent years, the specific RBPs that bind and control the regulated transcripts above typically remain unknown.

The RBPs comprise two main families in *T. brucei*; the RNA recognition motif (RRM) proteins account for 70–80 members, while the CCCH zinc-finger domain proteins, or ‘ZC3H’ proteins, comprise more than 130 members (17). In terms of developmental transitions; RBP10 promotes the bloodstream-form state, which is proposed to involve translational repression and destruction of insect stage specific mRNAs containing a UAU_6_ motif (18). REG9.1 mediates negative control in bloodstream-form cells of transcripts encoding transmission-associated surface proteins (19). RBP7 is required for quorum-sensing in bloodstream form cells (20) and RBP6, which is itself regulated by the double RNA-binding domain (DRBD) protein DRBD13 (21), promotes progression from the insect midgut stage to the insect salivary gland stage (22), possibly involving binding to a AU_3_AU_2_ motif (10). The small ZC3H proteins, ZFP1–3 (23–25) and the double ZC3H protein, ZC3H18 (26), are also involved in regulating differentiation from the bloodstream to insect stage. Other regulatory RBPs display developmental stage specific functions. For example, RBP42 binds within the coding sequence of mRNAs involved in energy metabolism in the insect stage (27) and the DRBD3 / polypyrimidine tract binding protein 1 (DRBD3/PTB1) binds and stabilizes a subset of developmentally regulated mRNAs encoding membrane proteins (28). In another study, however, DRBD3/PTB1 bound mRNAs encoding ribosomal proteins, translation factors and enzymes involved in energy metabolism (29). ZC3H11 binds and stabilizes, in an AU_2_-repeat dependent manner, transcripts involved in the heat-shock response in insect stage cells (30). Finally, the Pumilio family protein, PUF9, stabilizes transcripts during S phase (31). As above for regulated transcripts, there is also often a gap in understanding for the RBPs, based on failure to link specific regulatory RBPs to specific cohorts of regulated transcripts.

Control of the mitochondrial respiratome plays a central role in regulating energy metabolism in eukaryotes and this is also true in trypanosomatids (32). Mitochondrial metabolism differs substantially between developmental stages of *T. brucei*, for example (33), and reservoirs of *T. brucei* parasites in adipose tissue (34) and in skin (35), as well as circadian control of metabolism (36), suggest the need to continuously adapt the activity of the respiratome. The respiratome comprises five major complexes in the inner mitochondrial membrane; complex I, NADH:ubiquinone oxidoreductase; complex II, succinate dehydrogenase; complex III, cytochrome *c* reductase also known as the cytochrome *bc*1 complex; complex IV, cytochrome *c* oxidase; and complex V, the two-sector rotary F_o_F_1_ ATP-synthase. Complexes I, III and IV typically form respiratory supercomplexes (37) that, assisted by the mobile electron carriers ubiquinone and cytochrome *c*, couple proton pumping to electron transfer from NADH to oxygen to form water; protons are pumped from the matrix to the intermembrane space and generate a gradient (Δ*p*). Complex II also contributes to Δ*p* via reduction of ubiquinone. Complex V can then use the Δ*p* to produce ATP by oxidative phosphorylation. In bloodstream-form *T. brucei*, complex V generates Δ*p*, working in the opposite direction as a proton translocating ATPase (38).

Many fundamental discoveries regarding gene expression control in trypanosomatids emerged from studies on variant surface glycoproteins (VSGs) and our starting-point here was a genetic screen for factors involved in *VSG* gene silencing. The screen identified ZC3H39 and ZC3H40, and we used a combination of high-throughput and quantitative transcriptome, RNA–protein interaction, and proteomic analyses to characterize these proteins. We identify a respiratome regulon controlled by the conserved trypanosomatid ZC3H39/40 RBPs and we propose that this regulon facilitates rapid adaptation to environmental change.

## MATERIALS AND METHODS

### *T. brucei* strains

Bloodstream form *T. brucei* Lister 427 wild-type cells, 2T1 cells (39), and derivatives were grown in HMI-11 medium in a humidified incubator at 37°C and 5% CO_2_. Insect-stage *T. brucei* were grown in SDM-79 medium at 27°C. Genetic manipulation by electroporation using cytomix was carried out as described (40). Selection of recombinant bloodstream form clones was carried out by the addition of puromycin, phleomycin, hygromycin and blasticidin as required at 2, 2, 2 and 10 μg/ml, respectively; clones were subsequently maintained in 0.5, 1, 1 and 1 μg/ml of each antibiotic, respectively. Tetracycline at 1 μg/ml was used to induce RNAi knockdown and inducible expression. Cumulative growth analysis was carried out by counting cell density on a haemocytometer. Cultures were seeded at 1 × 10^5^ cells/ml, counted every 24 h and diluted as required. To generate the inducible ZC3H40 expressor strains, both bloodstream form and insect stage, the expression construct was introduced into a single *zc3h40* allele null strain prior to deletion of the second native *ZC3H40* allele, both latter steps carried out in the presence of tetracycline. Selection of recombinant insect stage clones was carried out by addition of puromycin, hygromycin and blasticidin at 2, 25 and 10 μg/ml, respectively; these clones were subsequently maintained in 1 μg/ml of each antibiotic. See the Supplementary Data File S1, Sheet S1 for further details.

### RNAi Target sequencing (RIT-seq) screen

The RIT-seq screen was carried out essentially as described (41), except that cells were selected on 100 μg/ml G418 for 7 days prior to extraction of genomic DNA. High-throughput sequencing was on a MiSeq platform (Illumina) at the Beijing Genomics Institute. Data were analysed as previously described (40). Briefly, reads were mapped to the *T. brucei* 927 reference genome (v6, tritrypdb.org) with Bowtie 2 (42) using the parameters –very-sensitive-local –phred33. Alignment files were manipulated with SAMtools (43) and a custom-script (40) and data were further assessed using the Artemis genome browser (44).

### Plasmid construction

RNAi target fragments for ZC3H39 (450 bp) and ZC3H40 (445 bp) were amplified and cloned into pRPa^iSL^ (45). Epitope tagging at the native locus was achieved using pNAT^X12myc^ or pNAT^XGFP^ to add *C*-terminal 12-myc or GFP tags, respectively (45). Fragments of the *C*-terminal 843 bp of ZC3H39 and 1136 bp of ZC3H40 were amplified and cloned into pNAT. A unique restriction enzyme site within the target fragment was used to linearize each plasmid prior to transfection (ZC3H39, XmaI; ZC3H40, BbsI). ZC3H39 and ZC3H40 gene disruption constructs were generated by cloning sequences amplified from upstream and downstream of the open reading frame to flank *BLA* and *PAC* (ZC3H40); and *BLE* and *HYG* (ZC3H39) selectable marker cassettes. Knockout cassettes were released by restriction enzyme digestion prior to transfection. For inducible expression, the ZC3H40 open reading frame (without the stop codon) was amplified and cloned into pRPa^ix6myc^ (45). See the Supplementary Data File S1, Sheet S1 for oligonucleotide sequences.

### Immunofluorescence microscopy

Immunofluorescence microscopy was carried out on fixed cells in 1% formalydehyde, dried onto slides in 1% BSA and, for internal antigens, permeabilized with 0.5% Triton X-100. Primary antibodies were used at 1:10 000 dilution (rat α-VSG-2 and rabbit α-VSG-6) and 1:5000 (mouse α-myc and rabbit α-GFP). Rhodamine or FITC conjugated secondary antibodies (Pierce) were used at 1:100. Cells were mounted in Vectashield (Vector Laboratories) containing DAPI (4′,6-diamidino-2-phenylindole). Images were captured using a Zeiss 710 microscope and were processed using Zen digital imaging suite and ImageJ.

### Protein blotting

Cells for western blotting analysis were lysed in Laemmli buffer, incubated at 98°C for 5 min and extracts were run into 10% SDS-PAGE gels. Transfer to nitrocellulose membrane (Protran 0.45 μM NC, Amersham) was carried out in Towbin buffer using a Bio-Rad semi-dry transfer system. Membranes were blocked and antibodies were incubated in 5% milk powder in PBS. Primary antibodies were used at 1:10 000 dilution (rabbit α-VSG-2 and rabbit α-VSG-6) and 1:5000 (mouse α-myc and rabbit α-GFP). The F1β-subunit of the mitochondrial ATP-synthase was detected using a polyclonal rabbit antiserum directed against the *Crithidia fasciculata* ATP synthase used at 1:500 dilution, which cross-reacts with the *T. brucei* orthologue (46). HRP conjugated secondary antibodies raised in rabbit and mouse (Bio-Rad) were used at 1:10 000. Blots were developed using an Amersham chemiluminescence kit according to manufacturer’s instructions.

### Protein immunoprecipitation

Cell lysates for co-immunoprecipitation analysis were prepared by harvesting 2 × 10^8^ cells by centrifugation at 900 × *g* for 10 min. The cell pellet was resuspended in 500 μl Trypanosome Lysis Buffer (20 mM Tris-Cl [pH 8.0], 400 mM NaCl, 1 mM EDTA, 0.5% NP-40, 10% Glycerol, 1 mM DTT and 1x Complete, EDTA-free protease inhibitor cocktail [Roche]). Cells were lysed by vortexing for 30 s followed by incubation on ice for 30 min. Cell debris was removed by centrifugation at 18 500 × *g* for 15 min at 4°C and supernatant (input) was incubated with 50 μl α-GFP coupled magnetic beads (Dynabeads) for a minimum of 1 h at 4°C with mixing. Beads were thoroughly washed with Trypanosome Lysis Buffer, resuspended in 50 μl Laemmli buffer (elution) and analysed by western blotting.

### Flow cytometry

Cells were harvested by centrifugation at 600 × *g* for 10 min at 4°C and washed with ice-cold PBS. Cell pellets were resuspended in ice-cold 70% (v/v) methanol in PBS and vortexed thoroughly. Cells were blocked in 50% FBS in PBS for 1 h at room temperature with mixing. Antibodies were incubated in 3% BSA in PBS for 1 h at room temperature with mixing. Wash steps and final resuspension were performed with 1% BSA in PBS. Samples were run on an LSR Fortessa (Beckton Dickinson) and data analysed using FlowJo.

### RNA-seq

Transcriptome analysis was carried out on a pair of wild-type Lister 427 clones and a pair of ZC3H40 RNAi knockdown clones grown in the absence of tetracycline or in the presence of tetracycline for 72 h. Total RNA was extracted using a Qiagen RNeasy kit according to the manufacturer’s instructions. Poly d(T) beads were used to enrich polyadenylated transcripts that were reverse transcribed before being sequenced on a HiSeq platform (Ilumina) at the Beijing Genomics Institute. Reads were mapped to a hybrid genome assembly as described previously (41). Briefly, the assembly consisted of the *T. brucei* 927 reference genome plus the bloodstream *VSG*-ESs and metacyclic *VSG*-ESs from the Lister 427 strain. Bowtie 2-mapping was with the parameters –very-sensitive –no-discordant –phred33. Alignment files were manipulated with SAMtools. Per-gene read counts were derived using the Artemis genome browser; MapQ, 0. Read counts were normalized using edgeR and differential expression was determined with classic edgeR. Reads Per Kilobase of transcript per Million mapped reads (RPKM) values were derived from normalized read counts in edgeR. Violin plots were generated using BoxPlotR (http://shiny.chemgrid.org/boxplotr/) also including cohorts of mitochondrial (47) and non-mitochondrial proteins (48).

### CLIP-seq

Cross-linking immunoprecipitation, followed by RNA-seq (CLIP-seq), was performed essentially as described previously (30). Briefly, 2 × 10^9^ cells were harvested by centrifugation at 900 × *g* for 15 min and resuspended in 10 ml Trypanosome Dilution Buffer (TDB; 80 mM NaCl, 20 mM Na_2_HPO_4_, 20 mM Glucose, 5 mM KCl, 1 mM MgSO_4_, 2 mM NaH_2_PO_4_, pH 7.4). Cross-linking with 400 mJ/cm^2^ (Uvitec) was performed in a 145 mm diameter Petri dish and cell pellets were frozen in liquid nitrogen. Cell pellets were defrosted in 500 μl ice-cold lysis buffer (10 mM NaCl, 10 mM Tris-Cl pH 7.5, 0.1% NP-50, 2× Complete, EDTA-free protease inhibitor cocktail [Roche], 40 U RNAsin [Promega], 8 mM Vanadyl ribonucleoside complexes [Sigma]). Cells were lysed by passing 14 times through a 27G needle at 4°C and cell debris was removed by centrifugation at 3500 × *g* for 8 min at 4°C. NaCl was added to a final concentration of 150 mM. 100 μl of α-GFP coupled magnetic beads (Dynabeads) were added and incubated for 2 h at 4°C with rotation. The (unbound) supernatant was retained and beads were washed extensively with ice-cold Immunoprecipitation buffer (IPP150: 150 mM NaCl, 10 mM Tris-Cl pH 7.5, 0.1% NP-40) and resuspended in 100 μl IPP150 (bound). Protease digestion of the unbound sample and the bound sample was performed by adding 0.2% SDS, 10 mM CaCl and 80 μg Proteinase K (Ambion) to 100 μl samples followed by incubation at 37°C for 30 min. RNA was extracted using Trifast (Peqlab) reagent according to manufacturer’s instructions and sent for sequencing on a HiSeq platform (Illumina) at the Beijing Genomics Institute. Reads were mapped and analysed as above for RNA-seq. Violin plots were also generated as above.

### Stable isotope labelling in cell culture (SILAC)

ZC3H40 knockdown, ZC3H39/40 double knockout or ZC3H40 inducible expresser cell lines and wild-type cell lines were labelled (all validated at >95% incorporation) with light (^12^C and ^14^N) or heavy (^13^C and ^15^N) labelled amino acids (Lys-8 ^13^C_6_^15^N_2_ and Arg-10 ^13^C_6_^15^N_4_) in HMI-11 media depleted of Lys and Arg (Gibco). Cells were mixed in a 1 heavy:1 light ratio and pelleted by centrifugation at 1000 × *g* for 10 min. Cell pellets were washed in PBS with 1× Complete, EDTA-free protease inhibitor cocktail (Roche) and lysed in 4% SDS, 100 mM Tris-Cl, pH 7.5. Cell lysates were run 2 cm into a 10% SDS-PAGE gel and fractionated into 8 slices. Slices were submitted to FingerPrints Proteomics (University of Dundee) for processing and analysis on an LTQ Orbitrap Velos Pro MS (Thermo Scientific) coupled to an UltiMate 3000 RSLC Nano UHPLC system. Peptide identification was performed using MaxQuant searching the *T. brucei* Lister427 annotated protein database at www.tritrydb.org.

### EC_50_ assays

Cells were seeded at 1 × 10^3^ cells/ml in 96-well plates in an oligomycin A (Sigma) 2-fold dilution series. After 72 h growth, 20 μl of 0.49 mM resazurin sodium salt (Sigma) in PBS was added to each well and the plates were incubated for a further 6 h. Fluorescence was determined using an Infinite 200 pro plate reader (Tecan) at an excitation wavelength of 540 nm and an emission wavelength of 590 nm. Data were analysed using Prism (GraphPad).

### Meme suite motif searching

Annotated 3′-UTRs in the *T. brucei* TREU927 reference genome sequence were downloaded from TriTrypDB (tritrypdb.org). Redundant UTRs and those shorter than 100 bp were removed from the dataset. The annotated 3′-UTR sequences of the respiratome cohort were used as the Primary sequences and the remaining 3′-UTRs were used as the Control sequences. The Meme Suite (meme-suite.org/tools/meme) was used for motif discovery using the parameters: discriminative mode, zero or one occurrence per sequence, search given strand only, minimum width 5, maximum width 15.

## RESULTS

### ZC3H39 and ZC3H40 knockdown leads to loss of *VSG*-silencing

**Figure 1.**
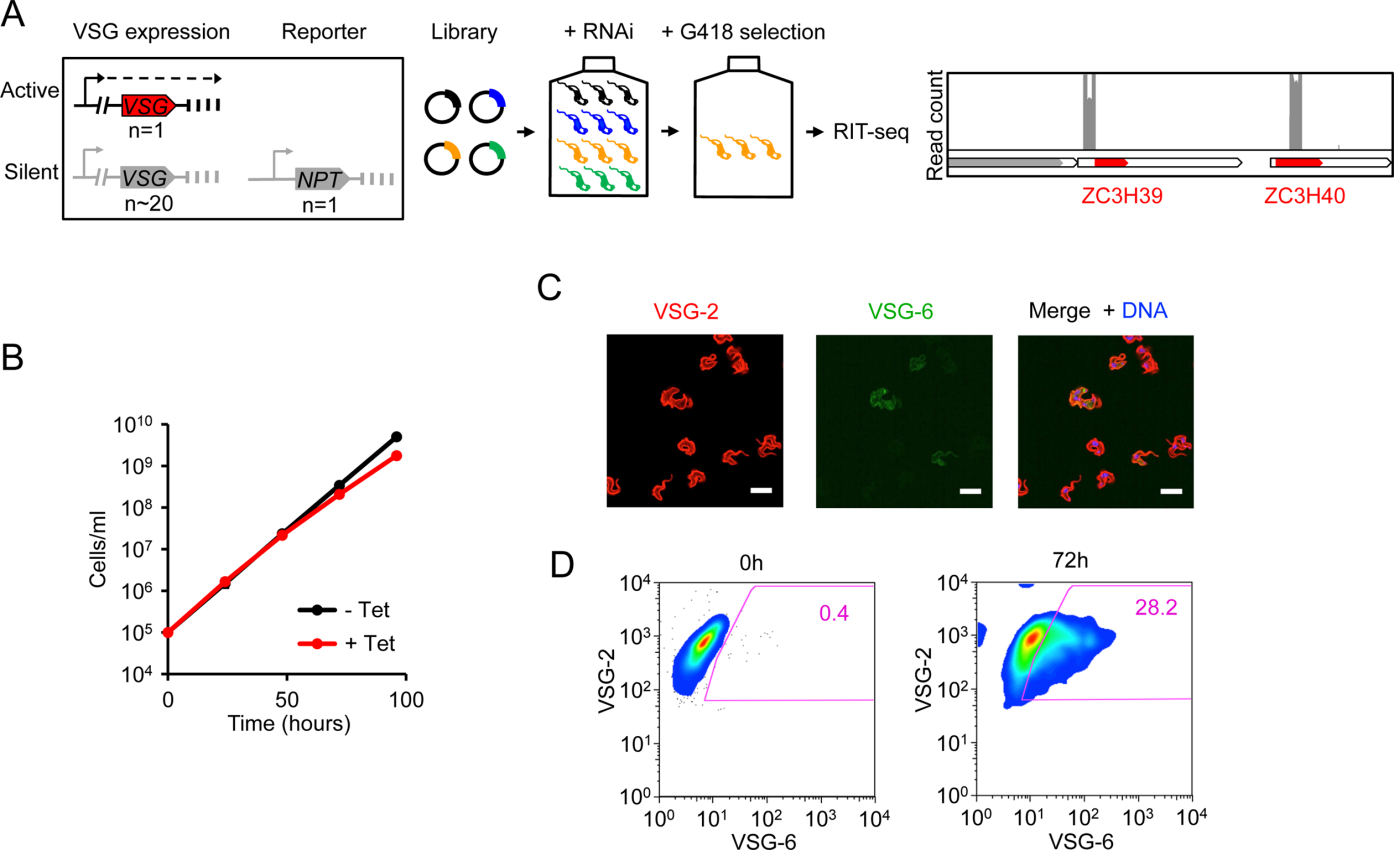
A genetic screen identifies a role for both ZC3H39 and ZC3H40 in *VSG*-silencing in bloodstream form *T. brucei*. (**A**) Schematic of reporter cell line, genomic screen selection overview and detail of ZC3H39 and ZC3H40 hits on chromomsome 10. (**B**) Growth analysis after induction of ZC3H40 knockdown (red). Data from technical replicates and two biological replicates. Error bars, SD; not readily visible as they are smaller than the symbols. (**C**) Immunofluorescent detection of VSG-2 and VSG-6 after ZC3H40 knockdown (72 h). DNA was counterstained with DAPI, scale bars 10 μm. (**D**) Flow cytometry analysis of surface VSG after ZC3H40 knockdown for 0 or 72 h, percentages of VSG-2 and VSG-6 positive cells within gated region (purple) are indicated.

We previously ran a high-throughput RNA interference (RNAi) screen for loss of telomeric gene silencing in bloodstream form *Trypanosoma brucei* in order to identify genes involved in controlling Variant Surface Glycoprotein (VSG) allelic exclusion (41); this screen revealed genes encoding VSG exclusion 1 (Tb927.11.16930, VEX1) and a telomere-associated protein (Tb927.6.4330). To identify further factors involved in *VSG* expression control, we ran a second high-throughput RNAi screen, using the system illustrated in Figure 1A, but this time with lower-stringency and a shorter timeframe of selection. Knockdowns that bring about derepression of the repressed telomeric *NPT* reporter and allow the cells to survive G418-selection are enriched in this screen. We used RNAi Target sequencing or RIT-seq (40) to identify hits and, once again, VEX1 emerged as a top hit. Among the top five hits, we also identified Tb927.10.14950. An RNAi target fragment mapped to the coding sequence of this gene, while a second independent fragment mapped to the 5′-untranslated region of the adjacent gene, Tb927.10.14930; these two genes on chromosome 10 encode the related putative RBPs, ZC3H40 and ZC3H39, respectively (Figure 1A, right panel). Thus, the genetic screen implicated both ZC3H genes in telomeric gene silencing control. Since gene expression control is primarily post-transcriptional in trypanosomatids, we considered these genes to be of particular interest.

To validate these hits, pairs of independent bloodstream form inducible RNAi knockdown strains, expressing VSG-2, and with *C*-terminal tagged native cognate ZC3H alleles were assembled and knockdown was induced using tetracycline. Growth was only minimally perturbed during ZC3H40 knockdown (Figure 1B), as predicted based on prior genome-wide fitness profiling (48). We assessed derepression of a silent VSG (VSG-6) in these strains, using immunofluorescence microscopy (Figure 1C). Efficient ZC3H40^myc^ knockdown was confirmed by protein blotting, which also confirmed robust VSG-6 derepression (Supplementary Figure S1A). Notably, knockdown lead to the appearance of cells expressing mixed VSG coats as determined by both immunofluorescence microscopy (Figure 1C) and flow cytometry (Figure 1D); double-positive cells increased from 1 ± 0.5% to 25 ± 3% (*n* = 3) following ZC3H40 knockdown, as determined by flow cytometry. Similar results were also obtained following ZC3H39 knockdown (Supplementary Figure S1B–D). Thus, these data validate both gene hits in the genome-scale screen for perturbed telomeric silencing and we conclude that both ZC3H39 and ZC3H40 are also required for robust silencing of telomeric *VSG* genes.

### ZC3H39 and ZC3H40 are related U-box ‘HC6H’ proteins

Orthologues of ZC3H39 and ZC3H40 were previously studied in another trypanosomatid, *Crithidia fasciculata*. These *Crithidia* proteins form an RNA-binding hetero-hexamer that was initially thought to control mRNA abundance during the cell-cycle (49); they were named ‘Cycling Sequence Binding Proteins’, CSBPA and CSBPB as a result. However, the mRNA transcripts under study were subsequently found to cycle even in the absence of these factors, and another distinct CSBP was identified, CSBPII, that binds the cycling mRNAs with higher affinity; CSBPII was indeed shown to control the cycling of these mRNA (50). Thus, since the putative ZC3H RBPs are not known to be required for cell cycle controls, we subsequently refer to them as ZC3H39/40 rather than CSBPA/B.

**Figure 2.**
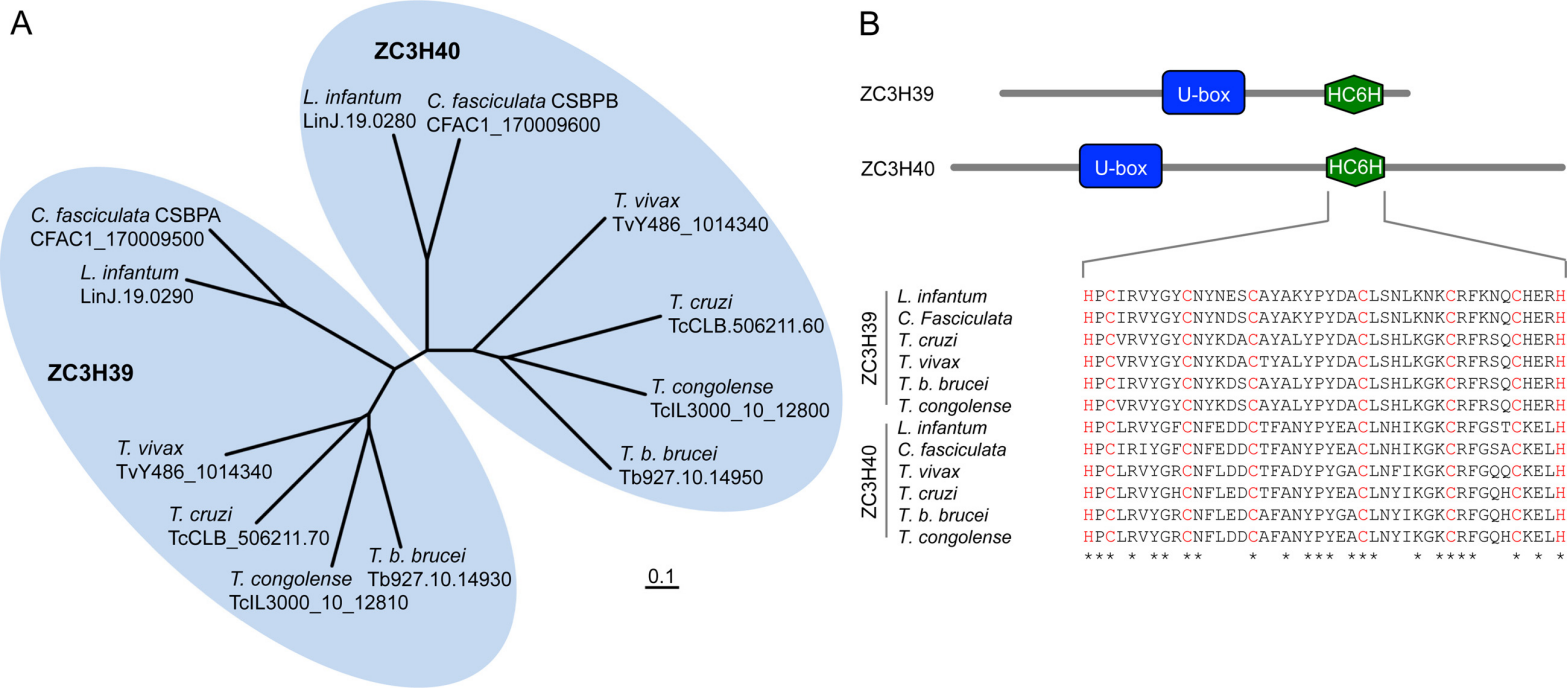
Phylogenetic analysis of ZC3H39 and ZC3H40 in the trypanosomatids. (**A**) Phylogenetic analysis of ZC3H39 and ZC3H40 in *T. b. brucei, T. congolense, T. vivax, T. cruzi, C. fasciculata* and *L. infantum;* sequences from tritrypbd.org. The unrooted neighbour-joining tree was produced using Clustal W2 and iTOL v3. (**B**) Schematic diagram of protein domains identified in *T. brucei* ZC3H39 and ZC3H40. The alignment shows residue conservation (*) in the HC6H ‘zinc finger’ domain. Putative zinc-coordinating cysteine and histidine residues are shown in red. Alignment produced using Clustal Omega.

Phylogenetic analysis of ZC3H39/40 orthologues from a number of trypanosomatids suggested that both genes were derived through duplication of a single gene and divergence in a common ancestor, with subsequent retention of both genes in all trypanosomatids analysed (Figure 2A), including several pathogens of humans and animals. Domain analyses revealed the Cys/His (C3H) motif in both putative proteins and, upon closer inspection, revealed a more extensive HC6H motif that was conserved across trypanosomatid ZC3H39 and ZC3H40 orthologues (Figure 2B). Both proteins also contain a putative U-box (Figure 2B); other U-box proteins constitute a family of ubiquitin-protein ligases (51).

### ZC3H39 and ZC3H40 form a cytoplasmic complex in *T. brucei*

**Figure 3.**
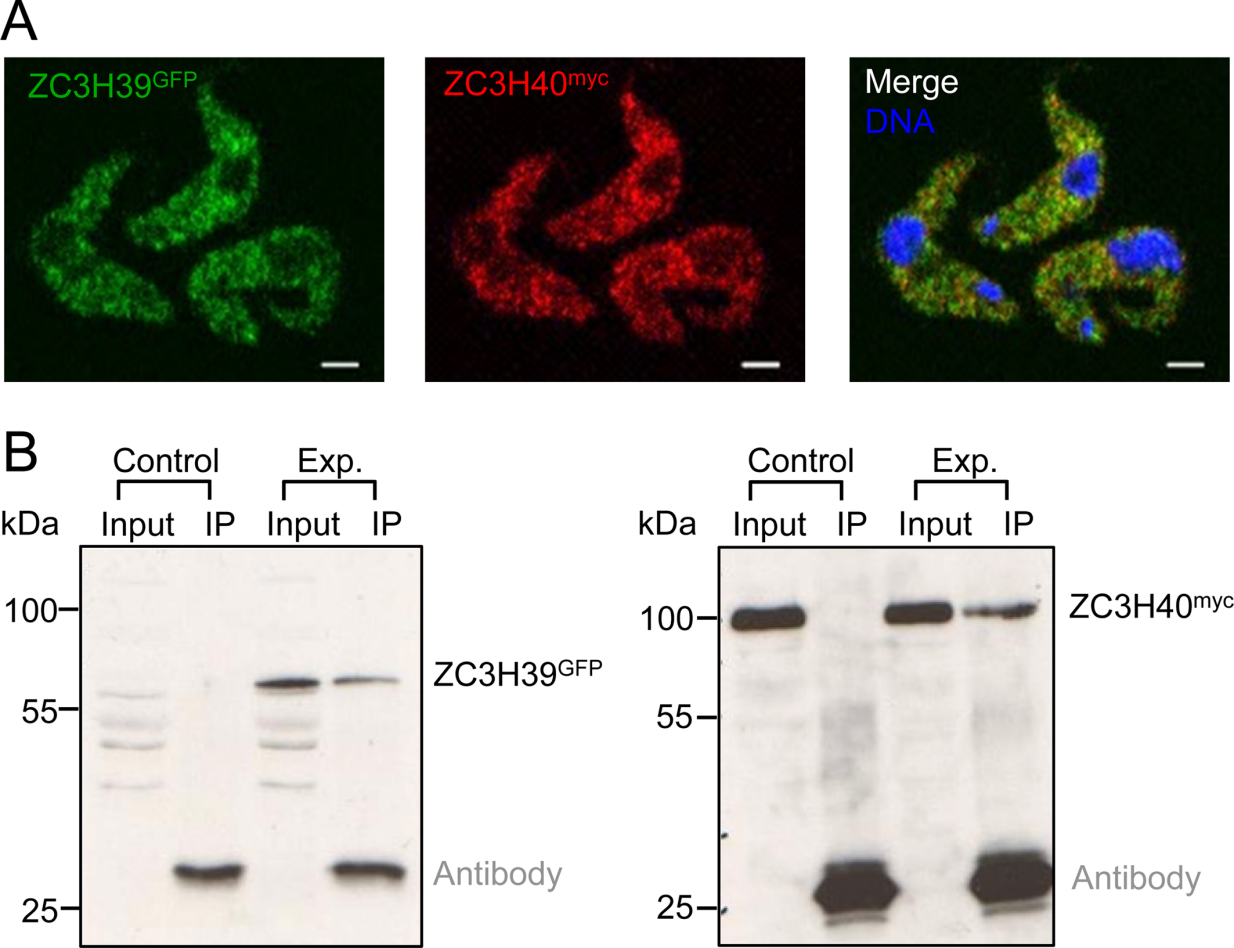
ZC3H39 and ZC3H40 form a complex in bloodstream form *T. brucei*. (**A**) Single plane confocal immunofluorescence microscopy detection of ZC3H39^GFP^ and ZC3H40^myc^. DNA was counterstained with DAPI; scale bars: 2 μm. (**B**) Co-immunoprecipitation analysis using strains expressing only ZC3H40^myc^ (Control) or ZC3H39^GFP^ plus ZC3H40^myc^ (Exp.). Immunoprecipitation (IP) was performed with mouse α-GFP antibody followed by protein blot analysis of input and IP samples with α-GFP antibody (left panel) and α-myc antibody (right panel). Antibody light chain signals are indicated.

Prior studies indicated that the *Crithidia* ZC3H39/40 orthologues form a hetero-hexameric complex (49). To explore ZC3H39 and ZC3H40 association in *T. brucei*, we first assessed bloodstream form strains expressing ZC3H39^GFP^ or ZC3H40^myc^ by confocal immunofluorescence microscopy. This revealed a punctate cytoplasmic localization for both proteins. We then assembled strains expressing both ZC3H39^GFP^ and ZC3H40^myc^ proteins and assessed the localization of both proteins simultaneously. Single plane confocal immunofluorescence microscopy analysis of these strains again revealed a punctate cytoplasmic localization for both proteins and substantial co-localization of ZC3H39^GFP^ and ZC3H40^myc^ (Figure 3A).

To further investigate the formation of a ZC3H39/40 complex in *T. brucei*, we used the dual-tagged strains for co-immunoprecipitation studies. The results indicated that these two proteins do indeed interact, consistent with formation of a ZC3H39/40 complex (Figure 3B). We also generated inducible ZC3H39 RNAi knockdown strains with a tagged allele of ZC3H40^myc^ and inducible ZC3H40 RNAi knockdown strains with a tagged allele of ZC3H39^GFP^. Protein blotting revealed that ZC3H40^myc^ was destabilized following ZC3H39 knockdown (Supplementary Figure S2, left-hand panel) and that ZC3H39^GFP^ was destabilized following ZC3H40 knockdown (Supplementary Figure S2, right-hand panel). Thus, ZC3H39/40 likely forms a bipartite cytoplasmic complex in *T. brucei*, as supported by immunofluorescence co-localization, co-immunoprecipitation and co-destabilization.

### ZC3H40 positively regulates respiratome expression

**Figure 4.**
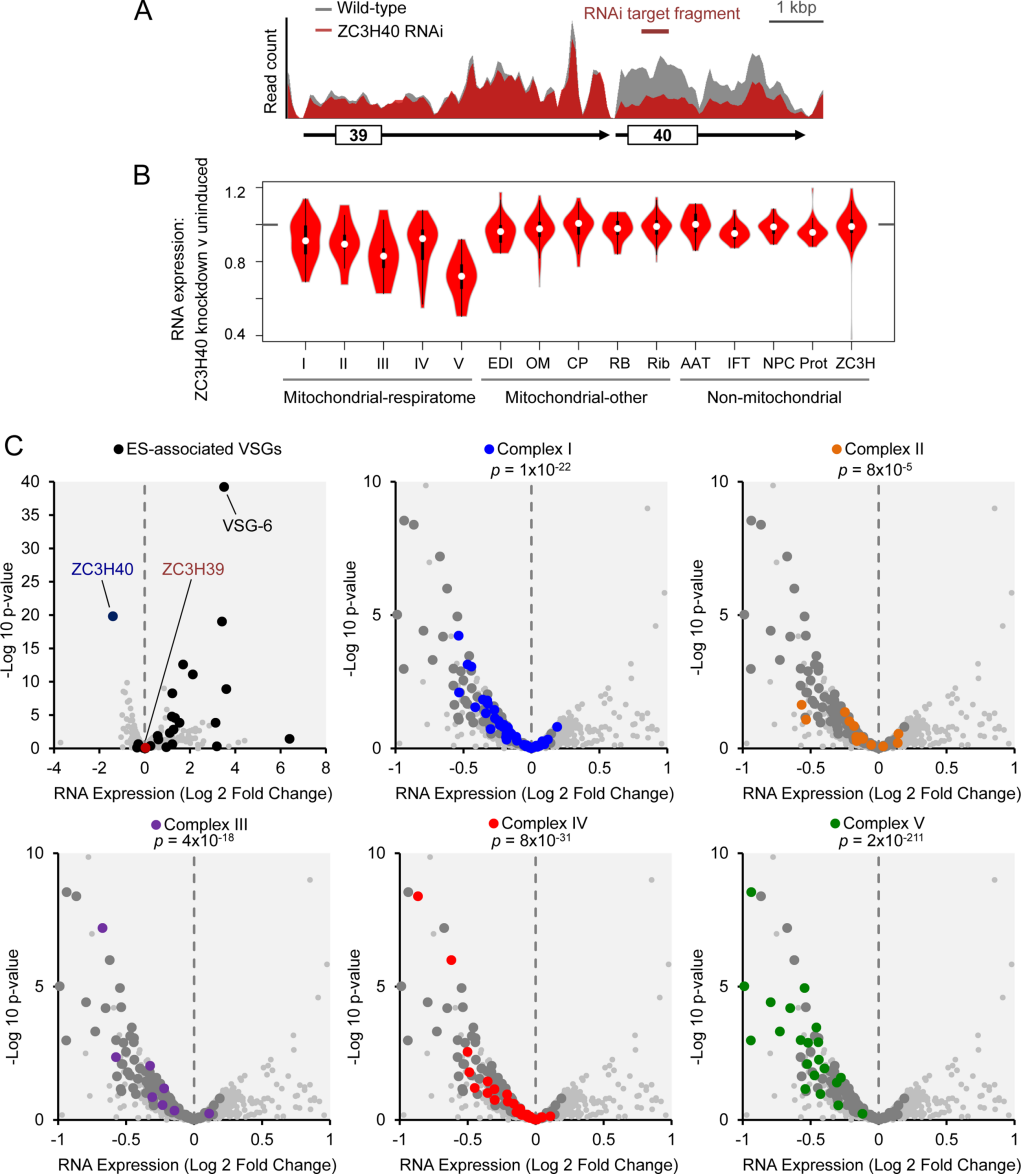
Transcriptome analysis following ZC3H40 knockdown in bloodstream form *T.brucei*. RNA-seq analysis following ZC3H40 knockdown (uninduced versus 72 h knockdown; also see Supplementary Data File S1, Sheet S2; Supplementary Figure S2). (**A**) The plot shows reads mapped across the *ZC3H39/40* locus in control cells (grey) or following ZC3H40 knockdown (red). Untranslated regions are indicated by black lines with arrowheads. (**B**) Violin plots for gene cohorts showing mRNA expression following ZC3H40 knockdown (RPKM). White symbols show the medians, box limits indicate the 25th and 75th percentiles and whiskers extend 1.5 times the interquartile range; AAT, amino acid transporters; CP, carrier proteins; EDI, editing complex; IFT, intraflagellar transport; NPC, nuclear pore complex; OM, outer membrane; Prot, proteasome; RB, RNA-binding complex; Rib, ribosome. (**C**) Volcano plots showing the full transcriptome (>7000 genes). Values are RPKM averages for two independent clones. ZC3H40 (blue) and ZC3H39 are shown in the top-left panel. ES-associated *VSGs*, all expression site associated *VSGs* are telomere-adjacent. In other panels, the larger grey data-points indicate the full respiratome set. *P*-values derived from chi-squared tests using transcripts down-regulated >20%.

Disruption of *VSG* silencing following ZC3H39/40 knockdown indicated that ZC3H39 and ZC3H40 are negative regulators of *VSG* genes. To ask whether ZC3H knockdown impacts the silencing of multiple *VSG* genes and/or other genes, we next used transcriptome analysis following ZC3H40 knockdown (Supplementary Data File S1, Sheet S2; Supplementary Figure S3). These analyses indicated highly specific knockdown of the ZC3H40 transcript and no significant impact on the ZC3H39 transcript (Figure 4A). Gene Ontology (GO) term analysis applied to the non-redundant RNA polymerase II transcriptome data revealed no enriched terms for significantly (*P* < 0.05) up-regulated genes (*n* = 16) but many enriched terms for down-regulated genes (*n* = 84); these GO-terms indicated specific down-regulation of genes encoding components of respiratome complexes (Supplementary Figure S4; Supplementary Data File S1, Sheet S3).

A number of proteome-based studies have clarified the composition of the respiratome complexes in *T. brucei* in recent years (32,47,52,53) and, using these datasets, we compiled a respiratome-set for this study (Supplementary Data File S1, Sheet S4); as in other eukaryotes, most of these components are encoded in the nuclear rather than mitochondrial genome in trypanosomatids. Analysis of this respiratome set and cohorts of ‘control’ transcripts encoding other mitochondrial or non-mitochondrial proteins (Supplementary Data File S1, Sheet S5) confirmed specific down-regulation of respiratome components following ZC3H40 knockdown (Figure 4B). Further analysis of the transcriptome revealed increased transcript abundance for many silent expression site-associated *VSG* genes, again confirming a role in telomeric *VSG* silencing (Figure 4C, top-left). In striking contrast, transcript abundance decreases for many genes encoding components of each respiratome complex (Figure 4C, other panels). For the respiratome complexes, we observed significant down-regulation in every case; complex I, NADH:ubiquinone oxidoreductase (*P* = 1 × 10^−22^); complex II, succinate dehydrogenase (*P* = 8 × 10^−5^); complex III, cytochrome *bc*1 complex (*P* = 4 × 10^−18^); complex IV, cytochrome *c* oxidase (*P* = 8 × 10^−31^) and complex V, F_o_F_I_ ATP-synthase (*P* = 2 × 10^−211^). Thus, ZC3H40, and likely the ZC3H39/40 complex (see above), negatively regulates RNA polymerase I transcribed *VSG* genes and positively regulates RNA polymerase II transcribed genes encoding respiratome components; this latter set includes 40 genes, distributed across ten different megabase chromosomes, that encode multiple components from each respiratome complex.

### The ZC3H39/40 complex binds transcripts encoding respiratome components

**Figure 5.**
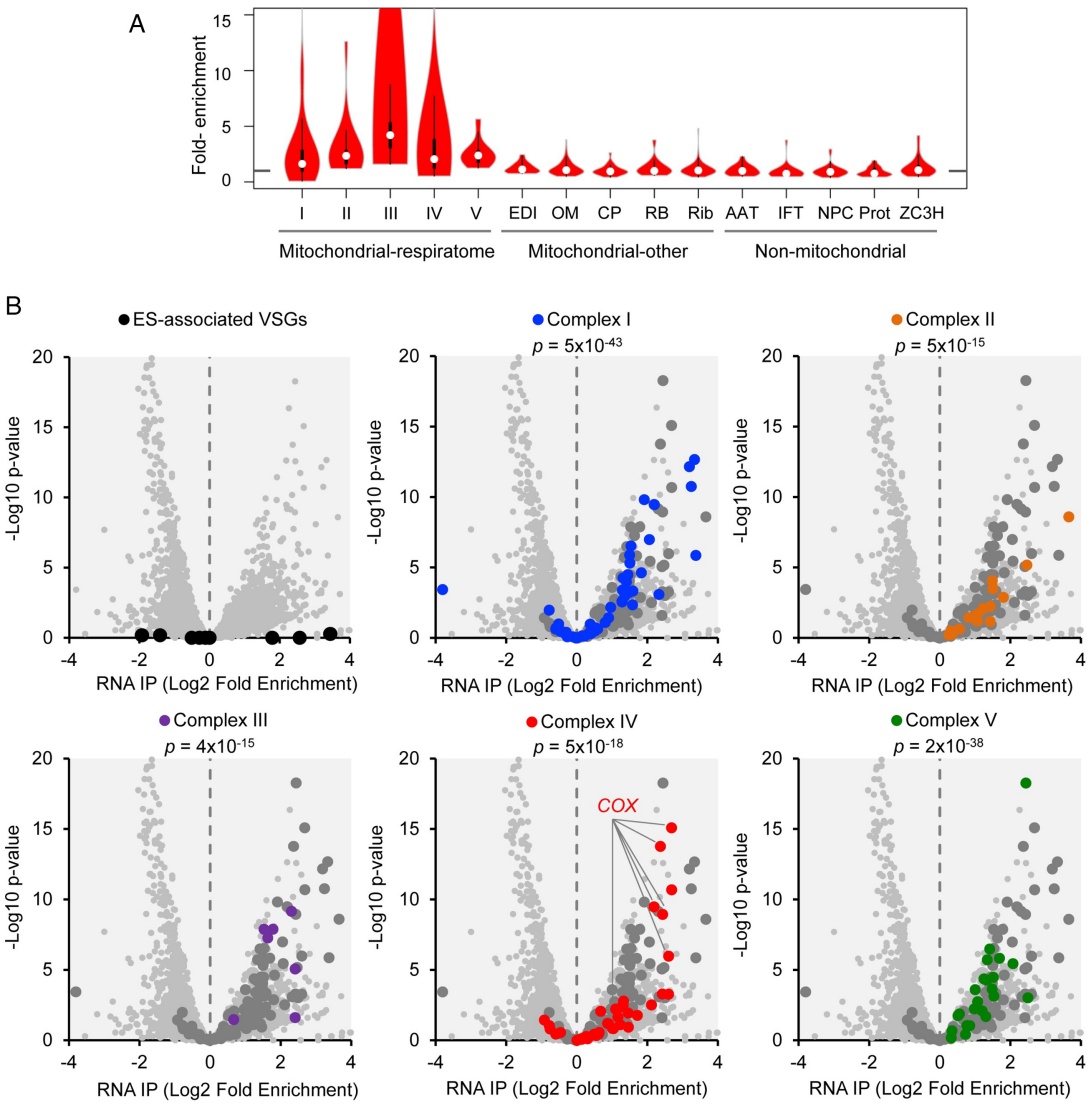
CLIP-Seq analysis with ZC3H39 and ZC3H40 in bloodstream form *T. brucei*. Cross-linking immunoprecipitation of ZC3H39^GFP^ and ZC3H40^GFP^ followed by RNA-seq analysis. (**A**) Violin plots for gene cohorts showing enrichment by CLIP-seq (RPKM); AAT, amino acid transporters; CP, carrier proteins; EDI, editing complex; IFT, intraflagellar transport; NPC, nuclear pore complex; OM, outer membrane; Prot, proteasome; RB, RNA-binding complex; Rib, ribosome. Other details as in Figure 4B. (**B**) Volcano plots for full transcriptome (>7000 genes). Values are averages for the ZC3H39^GFP^ and ZC3H40^GFP^ strains. *COX* transcripts are highlighted since they are particularly enriched among complex IV encoding transcripts. *P*-values derived from chi-squared tests using significantly (*P* < 0.01) enriched transcripts with fold-enrichment >2. Other details as in Figure 4C.

Transcriptome analysis indicated negative control of *VSG* genes and positive control of respiratome genes by the ZC3H39/40 complex. Notably, a genome-wide tethering screen in bloodstream form *T. brucei* initially reported positive control by ZC3H39 and ZC3H40 (54), while a subsequent screen reported negative control by the same two proteins (55). To determine whether native controls are directly mediated by RNA-binding, and to identify those transcripts bound by ZC3H39^GFP^ or ZC3H40^GFP^, we used cross-linking immunoprecipitation, followed by RNA-seq (CLIP-seq). As expected, since both proteins form a complex (see above), we observed very similar patterns of enrichment in the bound fraction relative to the unbound fraction for both ZC3H39 and ZC3H40 (Supplementary Data File S1, Sheet S6). Specifically, we observed enrichment of genes encoding respiratome complexes (Figure 5A), which matched the profile observed for down-regulated transcripts following ZC3H40 knockdown above (Figure 4B). Indeed, enrichment was specific for respiratome components and was not observed for cohorts of transcripts encoding other mitochondrial or non-mitochondrial proteins (Figure 5A). CLIP-seq data for silent *VSG* genes, and genes encoding components of each respiratome complex, are shown in Figure 5B. No *VSGs* were enriched (Figure 5B, top left). In contrast, transcripts encoding components of all five respiratome complexes were significantly enriched; complex I (*P* = 5 × 10^−43^), complex II (*P* = 5 × 10^−15^), complex III (*P* = 4 × 10^−15^), complex IV (*P* = 5 × 10^−18^) and complex V (*P* = 2 × 10^−38^). Thus, ZC3H39/40 binds and positively regulates respiratome transcripts. We conclude that ZC3H39/40 binding is associated with positive control of the respiratome, while negative regulation of *VSGs* is indirect.

**Figure 6.**
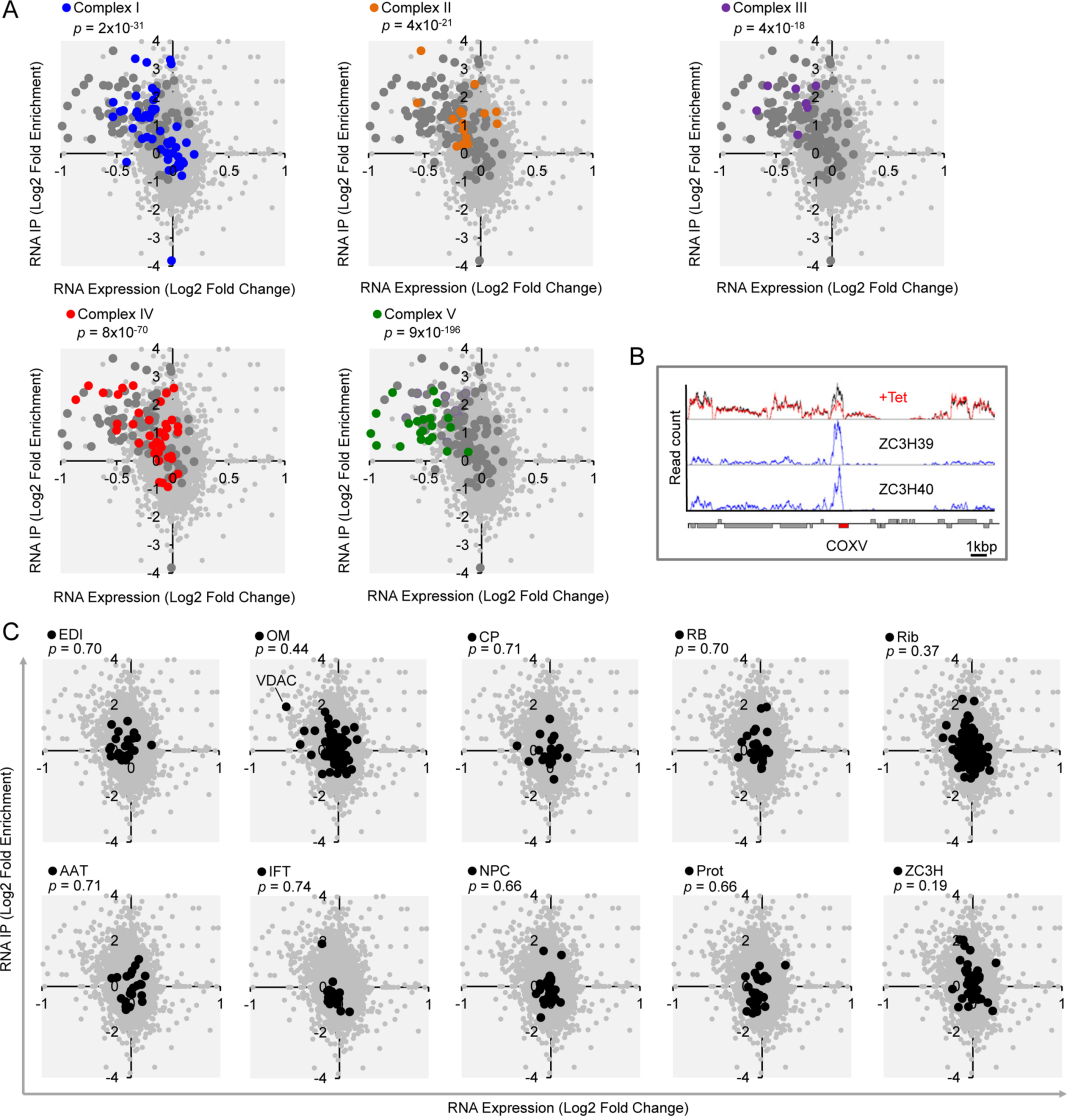
Stabilization of respiratome-encoding transcripts by ZC3H39/40 binding in bloodstream form *T.brucei*. (**A**) The plots show fold-change mRNA levels after ZC3H40 knockdown (RNA-seq) versus fold-enrichment after ZC3H40 RNA immunoprecipitation (CLIP-seq). The upper left-hand quandrants highlight respiratome-encoding transcripts that are bound by ZC3H40 and stabilized; the larger grey data-points indicate the full respiratome set. *P*-values derived from chi-squared tests using transcripts down-regulated >20% in the ZC3H40 knockdown and CLIP-seq enrichment of >2-fold. (**B**) The RNA-seq traces show read-count (RPM; Reads Per Million) at the *COXV* locus for ZC3H40 knockdown (top trace; black, uninduced; red, induced), ZC3H39 CLIP-seq (middle trace) and ZC3H40 CLIP-seq (bottom trace). (**C**) Plots as in panel (A) but for the additional mitochondrial (top row) and non-mitochondrial (bottom row) cohorts also analysed in Figures 4B and 5A; AAT, amino acid transporters; CP, carrier proteins; EDI, editing complex; IFT, intraflagellar transport; NPC, nuclear pore complex; OM, outer membrane; Prot, proteasome; RB, RNA-binding complex; Rib, ribosome. VDAC is indicated; see the text for more details.

We next combined the transcriptome and RNA-immunoprecipitation data to illustrate both binding to, and stabilization of, respiratome transcripts by ZC3H39/40 (Figure 6A). All five cohorts were significantly enriched in the upper left-hand quadrant of these plots, showing enrichment by immunoprecipitation and down-regulation following ZC3H40 knockdown; complex I (*P* = 2 × 10^−31^), complex II (*P* = 4 × 10^−21^), complex III (*P* = 4 × 10^−18^), complex IV (*P* = 8 × 10^−70^) and complex V (*P* = 9 × 10^−196^). An example locus encoding COXV, a component of complex IV, further illustrates specificity in terms of reduced abundance following ZC3H40 knockdown and enrichment by immunoprecipitation with either ZC3H39 or ZC3H40 (Figure 6B). We also show a series of similar plots for cohorts of other transcripts (Supplementary Data File S1, Sheet S5), none of which were significantly enriched in the upper left-hand quadrant (Figure 6C).

Illustrating the specificity of transcript binding and stabilization by ZC3H39/40, 80% of only 40 significantly bound (*P* < 0.01, >1.5× enriched) and stabilized (*P* < 0.05) transcripts encode annotated respiratome components; including multiple components from every complex, I–V (see Figure 6A). The remaining eight transcripts include those encoding the voltage-dependent anion channel (VDAC, Tb927.2.2510–2520; see Figure 6C), a membrane-associated mitochondrial protein (Tb927.11.2930) and several hypothetical proteins with ‘mitochondrion’ (Tb927.4.720, Tb927.5.2550, Tb927.10.2970, Tb927.11.2160) or ‘mitochondrial inner membrane’ (Tb927.10.4240) GO-term annotations. VDAC is the main metabolite channel in the outer membrane of *T. brucei* mitochondria (56), and may be coordinately regulated by ZC3H39/40. Notably, components of the branched electron transport chain (alternative oxidase, Tb927.10.7090; glycerol-3-phosphate dehydrogenase, Tb927.1.1130) and the alternative dehydrogenase (Tb927.10.9440) were neither significantly bound nor stabilized by ZC3H39/40. We conclude that *T. brucei* ZC3H39 and ZC3H40 form an RNA-binding complex that associates with and stabilizes multiple transcripts encoding each respiratome complex.

Since the results above reveal a respiratome regulon, represented by a well-defined cohort of transcripts, and regulated by interaction with a specific RBP; we took a bioinformatics approach to identify potential ZC3H39/40-binding motifs in the 5′- or 3′-untranslated regions (UTRs) of respiratome-encoding transcripts. Analysis of these UTRs revealed several significantly enriched, potential ZC3H39/40 RNA-binding motifs (Supplementary Table S1). These included a U-rich motif (*P* = 2 × 10^−20^) and an AG-rich motif (*P* = 6 × 10^−18^) in the 5′-UTR sequences; and a UC-rich motif (*P* = 2 × 10^−30^), an A-rich motif (*P* = 1 × 10^−28^), an AU-rich motif (*P* = 3 × 10^−23^) and a GA-rich motif (*P* = 5 × 10^−16^) in the 3′-UTR sequences.

### The ZC3H39/40 complex positively regulates respiratome protein expression

**Figure 7.**
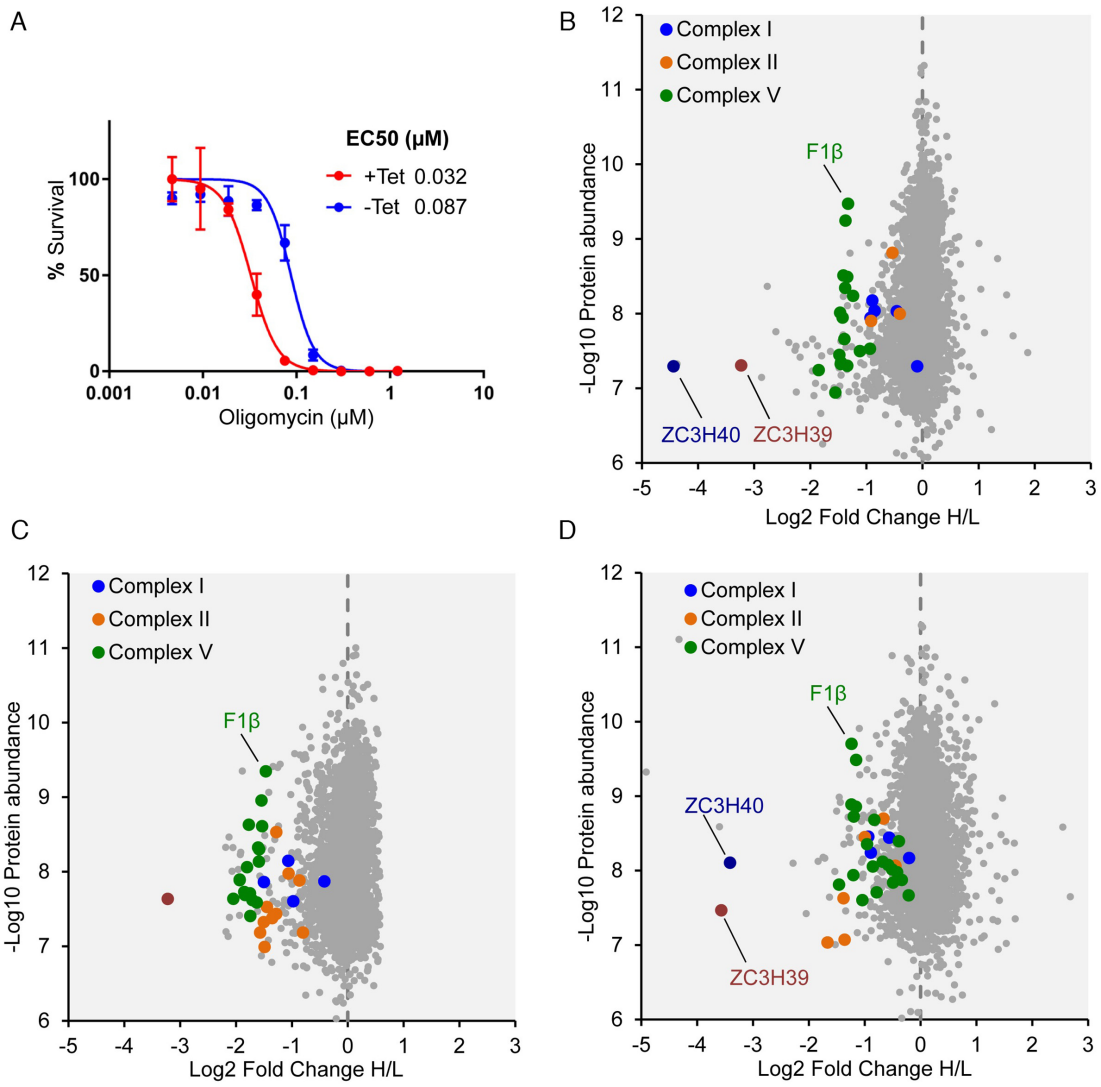
The ZC3H39/40 complex positively regulates respiratome protein expression in bloodstream form *T. brucei*. (**A**) Oligomycin dose–response curves before (-Tet) or after (+Tet) ZC3H39 knockdown. Error bars, SD from triplicate assays. (**B**) SILAC quantitative proteomic analysis; uninduced versus 72 h ZC3H40 knockdown. (**C**) SILAC quantitative proteomic analysis; induced versus uninduced ectopic ZC3H40^myc^. Tetracycline-inducible expression in a *zc3h40* null background with tetracycline removed 72 h prior to analysis. (**D**) SILAC quantitative proteomic analysis; *zc3h39 / zc3h40* double null versus wild type; incomplete isotope labelling accounts for the residual ZC3H39/40 signals.

Although highly significant, reduced abundance of transcripts encoding respiratome components was moderate (typically <2-fold) following ZC3H40 knockdown. We, therefore, assessed protein expression to determine whether ZC3H39/40 regulates the respiratome through control of translation; increased translation is known to increase mRNA half-life (6). First, we assessed the impact of the ZC3H39/40 proteins on complex V function using oligomycin, a specific inhibitor of the membrane-associated F_o_ sector of this two-sector rotary ATPase (the o indicates oligomycin sensitivity). If complex V was indeed depleted following ZC3H39/40 knockdown, we expected increased sensitivity to oligomycin and, consistent with our hypothesis, we observed almost 3-fold increased sensitivity to oligomycin (Figure 7A, ZC3H39; Supplementary Figure S5A, ZC3H40). We also assessed the expression of the F1β subunit of the ATP-synthase (complex V), using protein blotting. Again, consistent with our hypothesis, we observed substantially reduced abundance of this subunit following ZC3H40 knockdown (Supplementary Figure S5B).

Encouraged by these results, we next used stable isotope labelling in cell culture (SILAC) followed by mass-spectrometry to assess the abundance of a larger number of respiratome components (Supplementary Data File S1, Sheet S7). Quantitative proteomic analysis, comparing uninduced and induced ZC3H40 knockdown, revealed >8-fold down-regulation of ZC3H40 and, as expected (see Supplementary Figure S2), parallel down-regulation of ZC3H39 (Figure 7B). We also detected down-regulation of >20 respiratome components from complexes I, II and V (Figure 7B); while few components of complex III and IV were detected, consistent with relatively lower abundance in bloodstream form *T. brucei* (57–59). Thus, our quantitative proteomic analysis revealed significant depletion of respiratome proteins from complex I (*P* = 1.4 × 10^−13^), complex II (*P* = 2.3 × 10^−3^) and complex V (*P* = 1.4 × 10^−119^).

We used two further approaches to assess the impact of ZC3H39/40 on respiratome protein expression. First, we established strains expressing an ectopic and inducible copy of ZC3H40^myc^ in a null background (Supplementary Figure S5C). Second, we assembled a null strain for both ZC3H39 and ZC3H40 (Supplementary Figure S5D). SILAC-based quantitative proteomic analyses, comparing induced and uninduced ectopic ZC3H40 cells or wild-type and double *zc3h39/40* null cells, again revealed down-regulation of >20 respiratome components (Figure 7C and D; Supplementary Data File S1, Sheets S8 and 9); depletion of respiratome complexes was again highly significant in both experiments; complex I (*P* = 3.0 × 10^−12^ and *P* = 6.3 × 10^−11^, respectively), complex II (*P* = 7.9 × 10^−41^ and *P* = 3.5 × 10^−27^, respectively) and complex V (*P* = 1.3 × 10^−89^ and *P* = 1.2 × 10^−48^, respectively). A comparison of the RNA-seq (Figure 4C) and SILAC data (Figure 7B) for complex V components following ZC3H40 knockdown indicated ∼30% reduction in mRNA expression and ∼60% reduction in protein expression (Supplementary Figure S6), suggesting that both mRNA stability and translation are positively regulated.

Finally, we assessed the impact of ZC3H40 on respiratome protein expression in insect-stage *T. brucei* expressing an ectopic and inducible copy of ZC3H40^myc^ in a *zc3h40* null background. These cells displayed growth that was unperturbed (Supplementary Figure S7A) while ZC3H40^myc^ was efficiently depleted (Supplementary Figure S7B). SILAC-based quantitative proteomic analysis confirmed ZC3H40 depletion yet no major down-regulation of respiratome components (Supplementary Figure S7C; Supplementary Data File S1, Sheet S10); in this case, multiple components of all five respiratome complexes were detected, as expected in insect-stage cells (59). We conclude that ZC3H39/40 binding to respiratome-encoding transcripts facilitates positive control of respiratome expression in bloodstream form *T. brucei*. This conclusion is supported by increased sensitivity to oligomycin and quantitative proteomics with three distinct ZC3H39/40-disrupted strain-types. A distinct mechanism appears to sustain respiratome expression in the insect-stage.

## DISCUSSION

It is now widely accepted that post-transcriptional regulons make major contributions to coordinated gene expression control in eukaryotes, and in trypanosomatids in particular, yet few such regulons have been identified or characterized in any detail. Several studies have stalled in terms of linking specific regulatory RBPs to specific cohorts of regulated mRNAs, or vice versa. Other studies on RBP function have focussed on mRNA abundance rather than protein abundance measurements, meaning that translational control remains under-studied. We have used a combination of high-throughput genetic screening, transcriptomics, RNA–protein interactomic and quantitative proteomic analyses to identify ZC3H39/40, and to show that this bipartite RBP positively controls a respiratome regulon. Thus, regulation of polycistronically transcribed respiratome-encoding genes, distributed across all 11 megabase chromosomes in the *T. brucei* genome, is coordinated post-transcriptionally by ZC3H39/40. This likely facilitates rapid adaptation to environmental conditions.

To what extent is respiratome regulation likely required in *T. brucei*? The recent discovery of reservoirs of *T. brucei* parasites at sites outwith the bloodstream and central nervous system, namely adipose tissue (34) and the skin (35), suggests the need for adaptation to available carbon sources. Indeed, mammalian infective trypanosomes can use glucose for glycolysis, or glycerol for gluconeogenesis (60), and adipose tissue forms up-regulated pathways involved in lipid metabolism (34). Utilization of these different pathways may involve regulation of the respiratome. Indeed the respiratome is thought to be up-regulated in adipose tissue forms (34) and also in ‘stumpy’ cells that are pre-adapted for transmission to the insect vector (57,61). Respiratome regulation, therefore, may be important for growth in different host environments, at different times of the day due to circadian control of metabolism (36), and during the life cycle.

Proteome and transcriptome analyses in bloodstream form *T. brucei* revealed regulation of respiratome expression by ZC3H39/40. Translation increases mRNA half-life in *T. brucei* (6) and, consistent with this, respiratome mRNAs levels were moderately affected by ZC3H39/40 depletion. Many respiratome components are expressed at a relatively low level in cultured bloodstream form *T. brucei*, however, and at a higher level in insect stage cells (57,59). Perhaps surprisingly, our proteomic analyses indicated that the same ZC3H39/40 complex does not mediate substantial positive control of the respiratome in insect-stage cells. We suggest that additional factors contribute to these life cycle stage developmental differences. Indeed, previous work revealed a motif located in the 3′-UTR of transcripts encoding complex IV that is required for increased translation in insect stage *T. brucei* (12). Unfortunately, the RNA-binding proteins responsible have not been identified. In contrast, the ZC3H39/40 complex displays the capacity for positive control of all respiratome complexes in bloodstream form cells, but even in this life cycle stage it seems likely that an additional factor can exert negative control, on components of complexes III and IV in particular.

The presence of a cytoplasmic ZC3H39/40 complex in *T. brucei* was demonstrated by immunofluorescence co-localization, co-immunoprecipitation and co-destabilization. The *ZC3H39* and *ZC3H40* genes were likely duplicated in a common ancestor and subsequently diverged, whilst being retained in all trypanosomatids investigated. Thus, a hetero-oligomeric ZC3H39/40 complex, as described in *Crithidia* (49), may be critical to function in multiple trypanosomatids. Both ZC3H39/40 proteins contain a HC6H motif and a U-box, the latter typically found in ubiquitin-protein ligases (51). Another *T. brucei* gene encodes an HC6H motif and a UBA domain (Tb927.9.3460, ZC3H27) and two others encode an HC6H domain (Tb927.7.2580, ZC3H19; Tb927.4.3540). *T. brucei* ZFP2 also contains a WW domain typically found in E3 ubiquitin ligases (23). RNA-binding ubiquitin ligases are also found in other eukaryotes, but the link to mRNA regulation currently remains unclear (62).

RBPs can be regulated by post-translational phosphorylation. For example, phosphorylation of human Y-box binding protein-1, which may also control a respiratome regulon (63), releases this factor from mRNA and activates translation (64). Yeast Puf3, which can positively or negatively regulate mitochondrial proteins, is also dependent upon its phosphorylation status (65). Notably, ZC3H40 displays a major cluster of 12 Ser/Thr phosphorylated sites in bloodstream form cells, between residues 322–363 (66). In the case of ZC3H39/40, we speculate that these two closely related proteins function as a regulatory pair, whereby the activity of one component is regulated by the other as part of the complex; this may be similar to enzyme–prozyme pairs, such as the major protein arginine methyltransferase in *T. brucei*, PRMT1, for example (67). Thus, we favour a model whereby ZC3H39/40 interactions vary depending upon the environmental conditions, allowing responsive control of respiratome expression. At this stage though, due to the co-destabilzation of ZC3H39 and ZC3H40 after depletion of either protein in bloodstream form cells, we have not yet been able to dissect individual functions for each of the two components.

Studies in another trypanosome, *T. cruzi*, suggest a related function for ZC3H39. In this case, this *T. cruzi* RBP was found, under stress conditions, to interact with mRNAs encoding the cytochrome *c* oxidase complex, and to negatively regulate expression (68); these authors also identified an A_3_CA_2_ ZC3H39 binding-motif in the 3′-UTR. We identified potential ZC3H39/40-binding motifs within 5′-UTR or 3′-UTR sequences of respiratome mRNAs. Computational approaches have been applied to the identification of putative linear and structural regulatory elements within *T. brucei* UTRs (10). However, the identification, and our understanding, of UTR regulatory elements in eukaryotes remain challenging and rudimentary, respectively (2). Further work will be required to explore ZC3H interactions with potential linear or structural motifs, or potentially within nascent polypeptides.

Since most respiratome components are encoded in the nuclear genome, respiratome assembly requires cytoplasmic translation and mitochondrial import (69). In eukaryotes, these processes appear to be coordinated, with mitochondrial proteins often translated at the mitochondrial membrane (70). Indeed, RBPs are thought to regulate the import of respiratome components in diverse eukaryotes (70,71). For example, the human Y-box binding protein-1 noted above is associated with respiratome encoding mRNAs that are polysomal and bound to mitochondria (72). Yeast Puf3, also noted above in association with respiratome encoding mRNAs, is also localized to mitochondria (73). Thus, despite the divergent import machinery (69), RBPs may control the import of respiratome components in trypanosomatids. It is notable in this regard that mitochondrial import does not always require a conventional cleaved presequence in *T. brucei*, as demonstrated for a respiratome complex IV component (74).

In conclusion, we report a major respiratome regulon in trypanosomes, under the direct control of the ZC3H39/40 RNA-binding proteins. Our findings also establish an intriguing, yet indirect, link between the respiratome regulon and *VSG* expression control, which may reflect an ability to adjust, or even switch VSG expression in response to environmental cues; via the inositol phosphate pathway, for example (75). In terms of direct control, the ZC3H39/40 RNA-binding complex specifically coordinates the expression of the respiratome, which we suggest facilitates rapid adaptation to environmental change. To our knowledge, the respiratome represents the largest linked cohort of genes that are coregulated by a known RBP in any trypanosomatid.

## DATA AVAILABILITY

The sequence data reported in this paper have been deposited in the European Nucleotide Archive, www.ebi.ac.uk/ena (accession no. PRJEB30784).

The proteomic data reported in this paper have been deposited in the PRIDE Archive, www.ebi.ac.uk/pride/archive/ (accession no. PXD012450).

## Supplementary Material

gkz455_Supplemental_Files

## Notes

Present address: Lucy Glover, Trypanosome Molecular Biology, Department of Parasites and Insect Vectors, Institut Pasteur, 25-28 Rue du Docteur Roux 75015, Paris, France.

Present address: Sebastian Hutchinson, Trypanosome Cell Biology Unit, INSERM U1201, Department of Parasites and Insect Vectors, Institut Pasteur, 25-28 Rue du Docteur Roux 75015, Paris, France.
