# Supplementary material for: A post-transcriptional respiratome regulon in trypanosomes
Source: Nucleic Acids Res. 2019 May 25;47(13):7063–77. doi: 10.1093/nar/gkz455 (PMC6648352; doi:10.1093/nar/gkz455)
Supplement: gkz455_Supplemental_Files [file gkz455_supplemental_files.zip › Trenaman Supplementary Data.pdf]

Supplementary Data for:

A post-transcriptional respiratome regulon in trypanosomes

Anna Trenaman, Lucy Glover, Sebastian Hutchinson and David Horn

This PDF file includes:

Supplementary Figures S1-S7 and Table S1

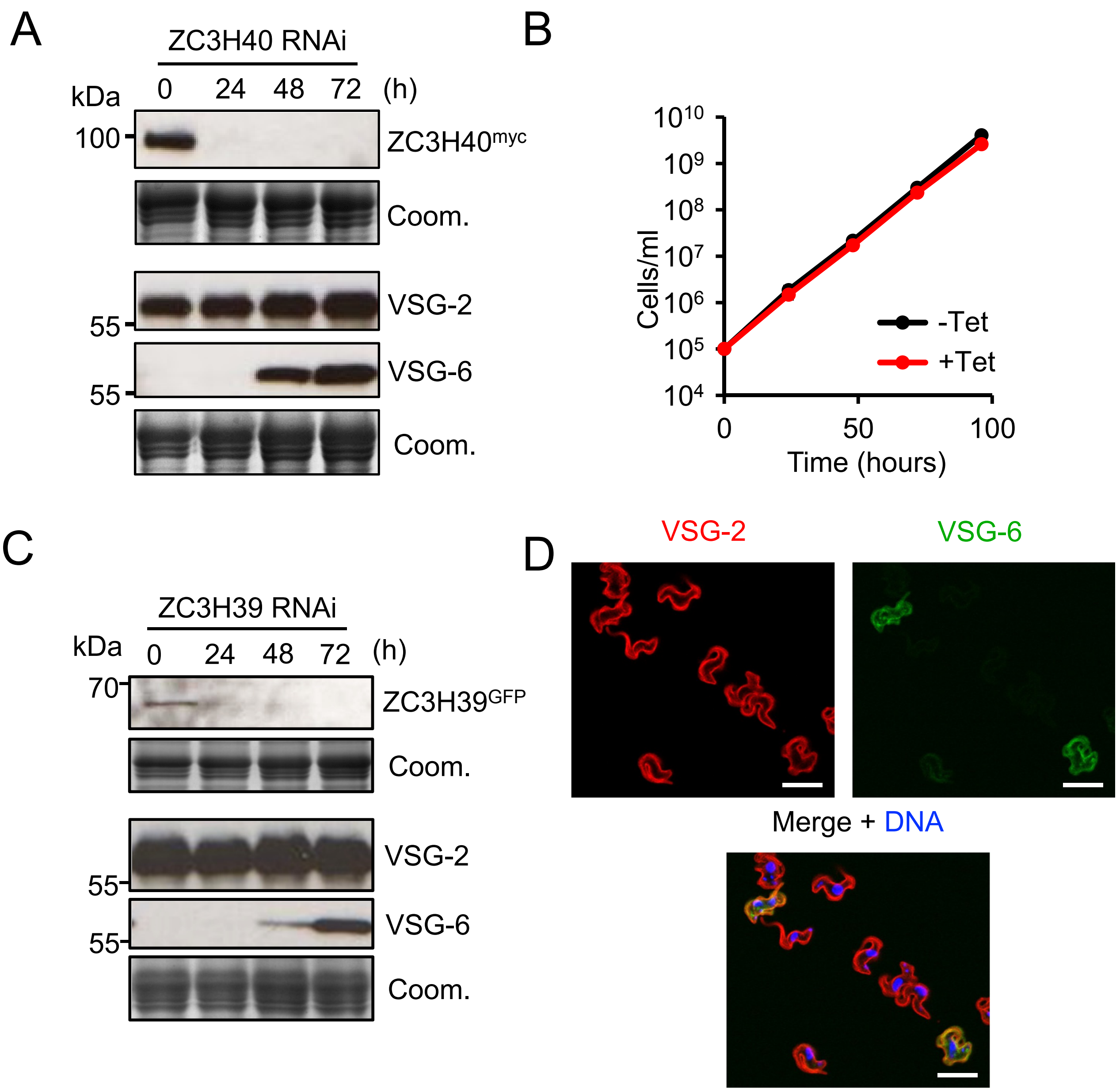

Supplementary Figure S1. ZC3H39/40 knockdown by RNAi. (**A**) Protein blot analysis after ZC3H40 knockdown shows reduction of ZC3H40^{myc} (predicted mass, 64 kDa) and VSG-6 derepression. The Coomassie-stained gels serve as sample loading controls. (**B**) Growth analysis after induction of ZC3H39 knockdown (red). Data from technical replicates and two biological replicates. Error bars, SD; not readily visible as they are smaller than the symbols. (**C**) Protein blot analysis after ZC3H39 knockdown shows reduction of ZC3H39^{GFP} (predicted mass, 59 kDa) and increased VSG-6 expression. The Coomassie-stained gels serve as sample loading controls. (**D**) Immunofluorescence detection of VSG-2 and VSG-6 after ZC3H39 knockdown (72 h). DNA was counterstained with DAPI, scale bars 10 µm.

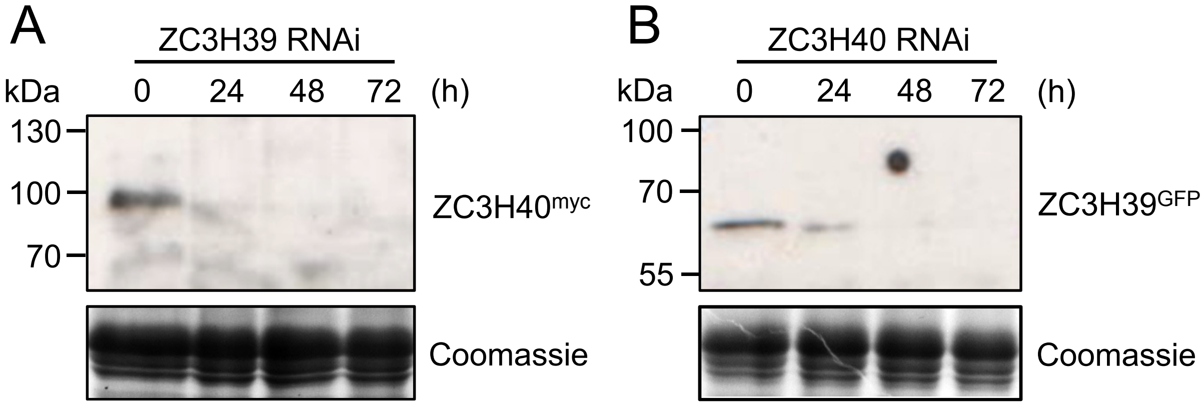

Supplementary Figure S2. Protein blot analysis after ZC3H39/40 knockdown. ZC3H40^{myc} expression after ZC3H39 knockdown (left panel), and ZC3H39^{GFP} expression after ZC3H40 knockdown (right panel). Coomassie-stained gels serve as sample loading controls.

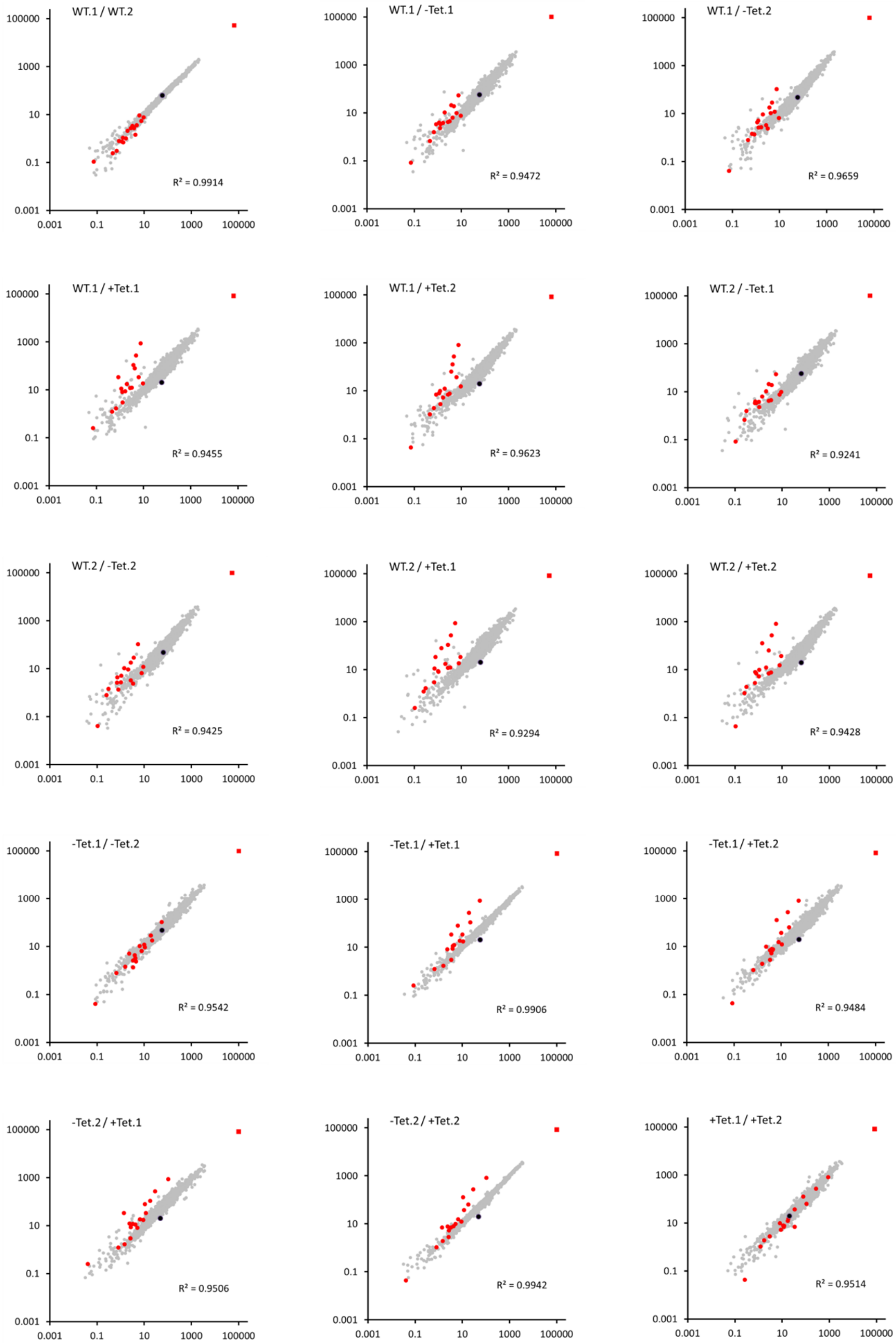

Supplementary Figure S3. RNA-seq analysis following ZC3H40 knockdown by RNAi. Pairwise comparisons. Red, expression-site associated *VSGs*; black, *ZC3H40*. Pearson correlation coefficients are indicated.

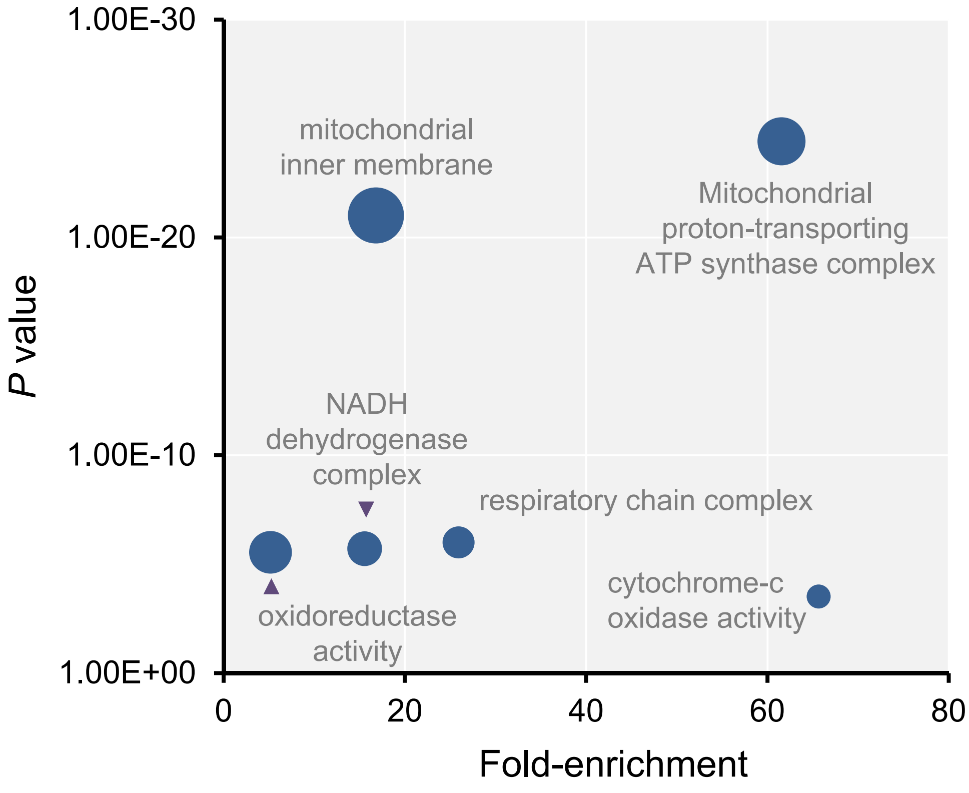

Supplementary Figure S4. GO-term analysis. GO-term profile for down-regulated transcripts (all 84 with *P* <0.05). All results shown are >2-fold enriched, represented by a minimum of 2 genes and with *P* <0.0001 (see Supplementary Data File, sheet 3).

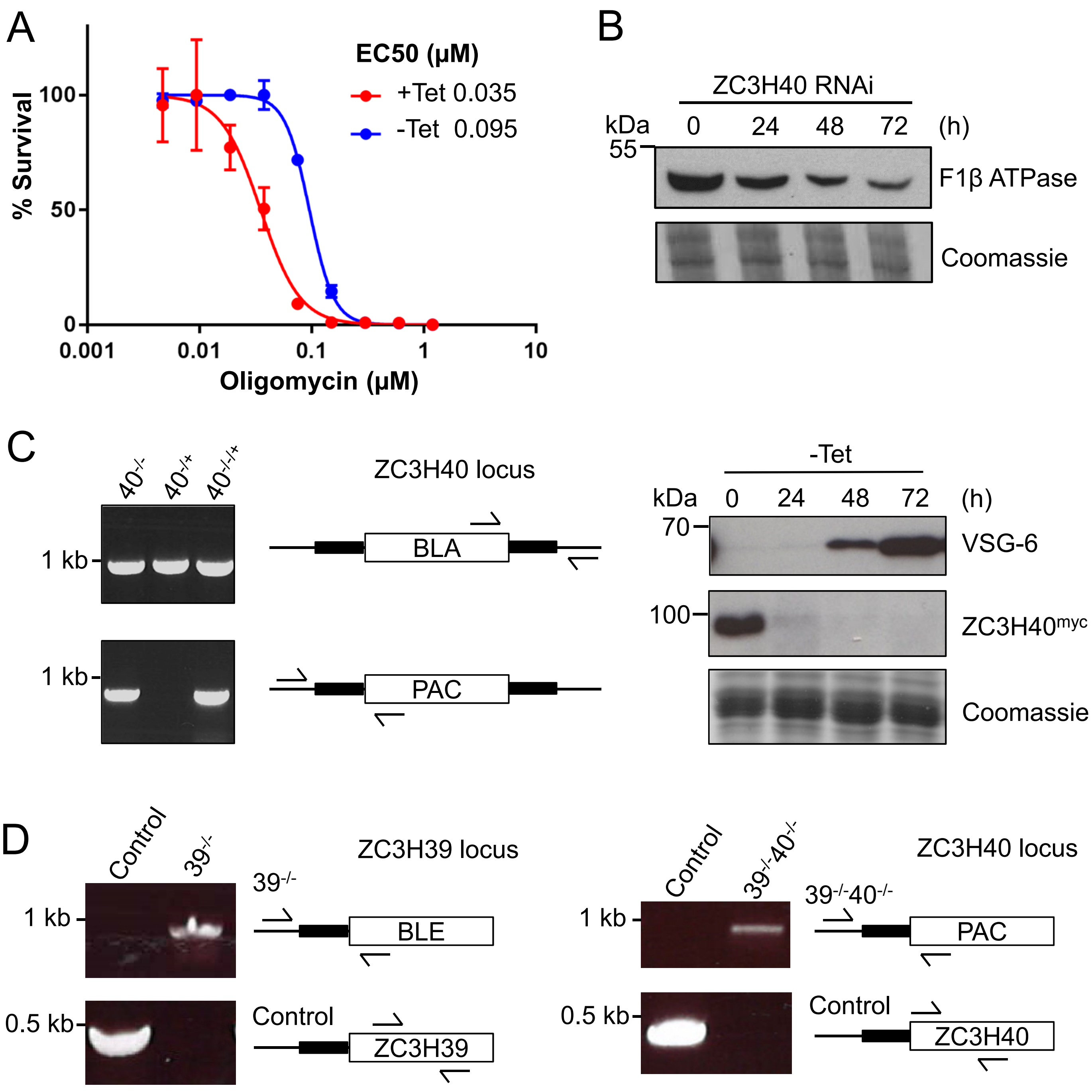

Supplementary Figure S5. Analysis of ZC3H40 knockdown, conditional ZC3H40^{myc} and *zc3h39/40* double null strains. (**A**) Dose response curve for ZC3H40 knockdown cells in the presence of oligomycin. Error bars, SD from triplicate assays. (**B**) Protein blotting showing ATP-synthase F1β subunit expression during ZC3H39 knockdown. The Coomassie-stained gel serves as a sample loading control. (**C**) PCR analysis of ZC3H40^{myc} conditional overexpression in a *zc3h40* null background (left panel, 40$^{-/-/+}$); *zc3h40* null (40$^{-/-}$) and single-allele null (40$^{-/+}$; *BLA*) strains serve as controls. The PCR primers were used as shown. Protein blot analysis (right panel). Tetracycline was removed from a ZC3H40^{myc} conditional overexpression strain for 0 to 72 h. The Coomassie-stained gel serves as a sample loading control. (**D**) PCR analysis of a *zc3h39* null strain (left panel) and a subsequently derived *zc3h39/40* double null strain (right panel). The PCR primers were used as shown.

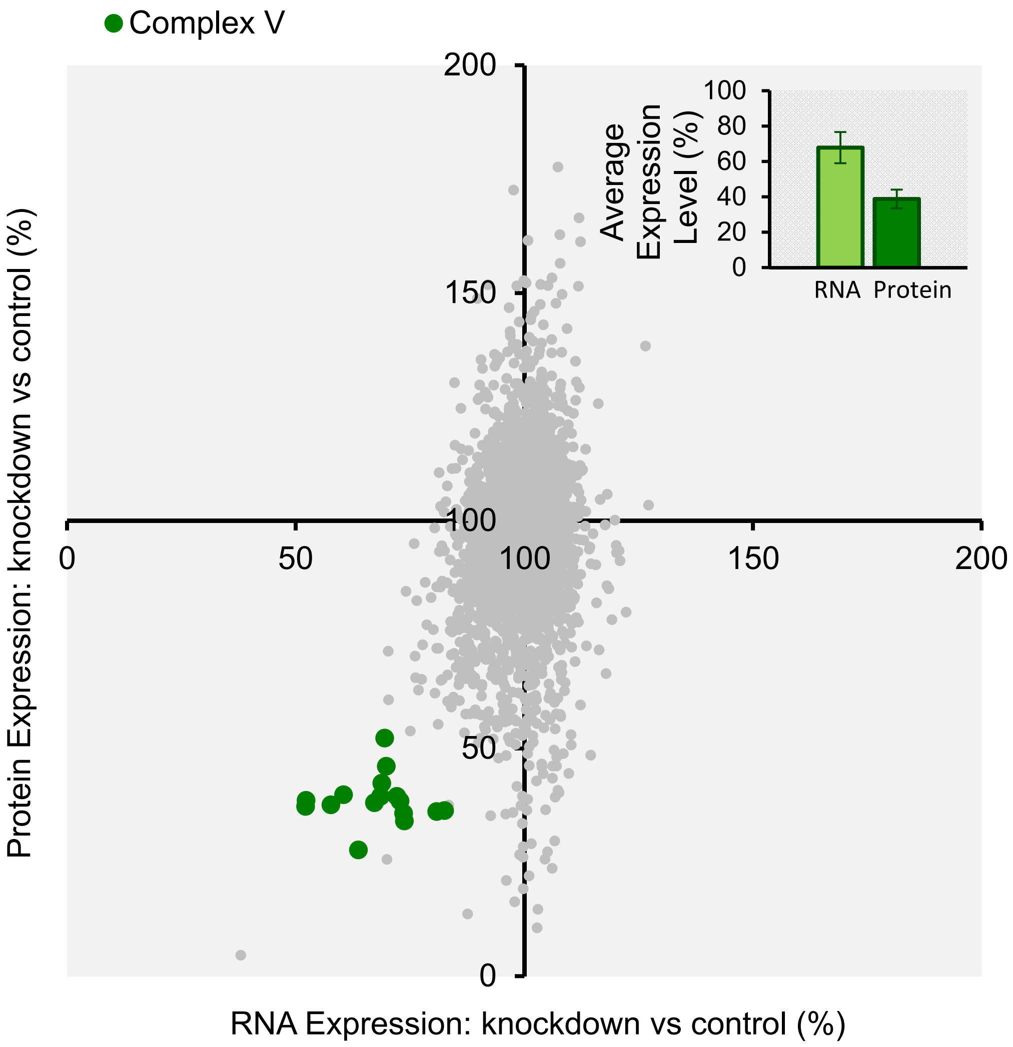

Supplementary Figure S6. A comparison of the RNA-seq (Figure 4C) and SILAC data (Figure 7B) for complex V component expression following ZC3H40 knockdown.

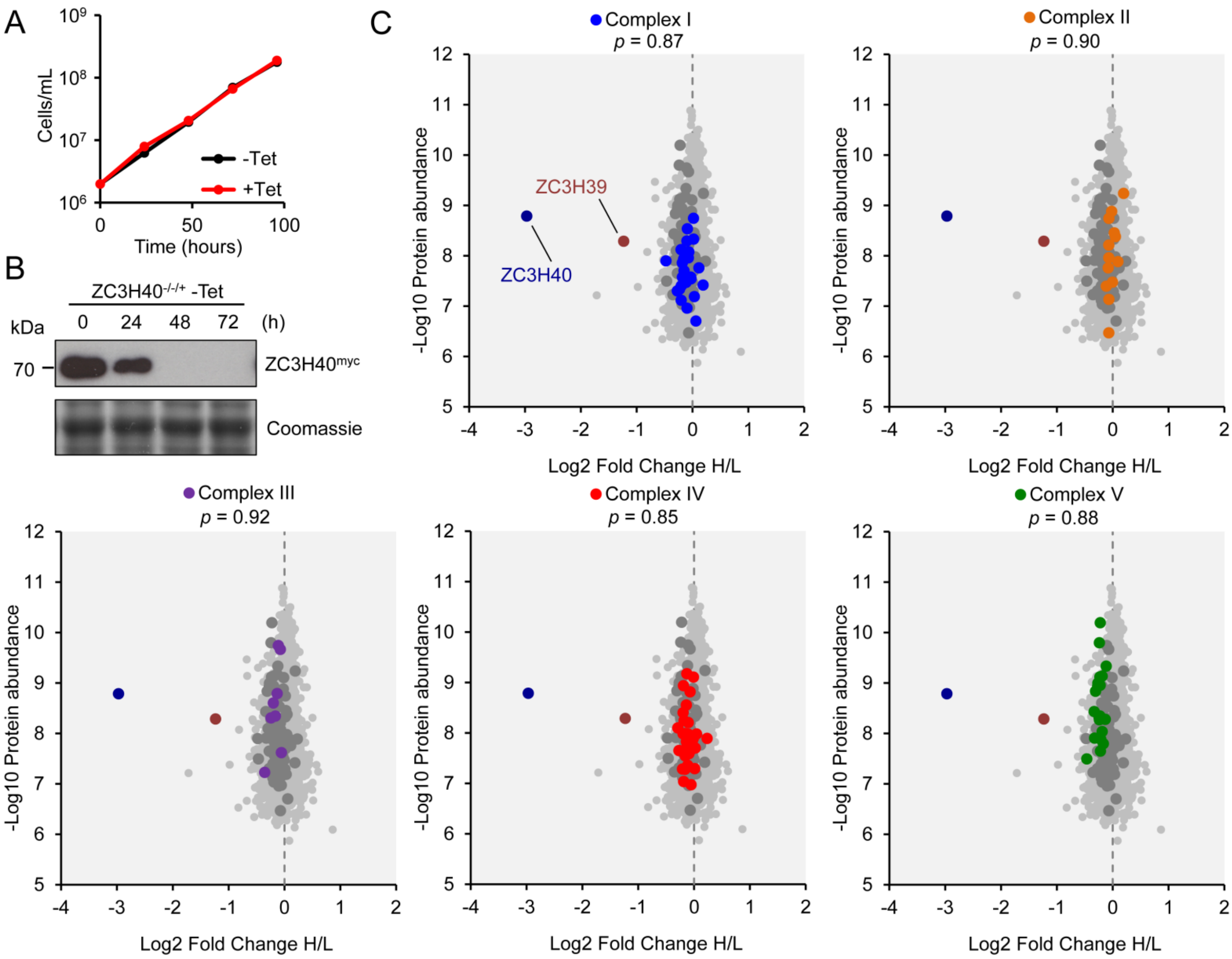

Supplementary Figure S7. Analysis of conditional null ZC3H40^{myc} insect stage *T. brucei* strains. (**A**) Growth analysis following inactivation of ZC3H40^{myc} (black). Data from technical replicates and three biological replicates. Error bars, SD; not visible as they are smaller than the symbols. (**B**) Protein blot analysis of ZC3H40^{myc} conditional overexpresser cells following removal of tetracycline. The Coomassie-stained gel serves as a sample loading control. (**C**) SILAC quantitative proteomic analysis; induced v uninduced ectopic ZC3H40^{myc}.

UTR	Logo	E-value	Site count
5’	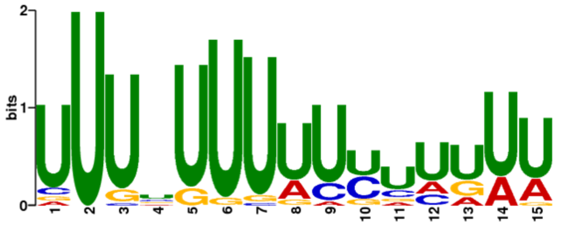	2.1e-20	39
5’	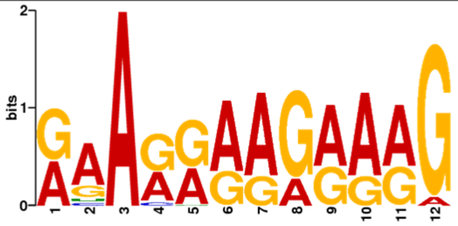	5.7e-18	49
3’	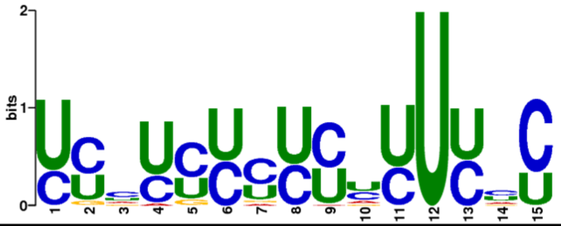	2.1e-30	77
3’	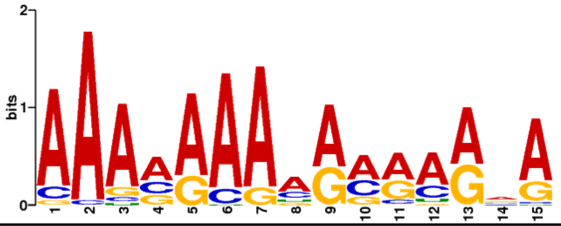	1.3e-28	60
3’	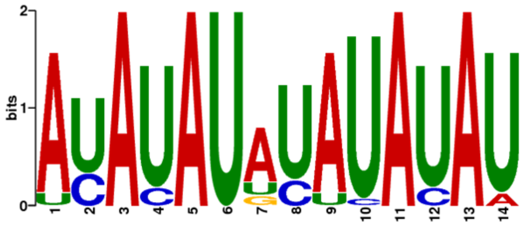	3.1e-23	23
3’	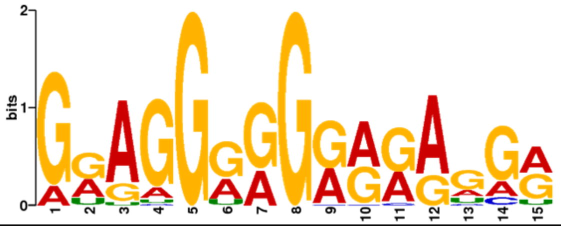	5.4e-16	46

Supplementary Table S1. Respiratome-enriched motifs identified using Meme Suite.
Sequence motif logos identified in the 5’ (top 2 motifs) and 3’ (bottom 4 motifs) untranslated regions of respiratome encoding transcripts.
